# Extracellular vesicles from virulent *P. brasiliensis* induce TLR4 and dectin-1 expression in innate cells and promote enhanced Th1/Th17 response

**DOI:** 10.1080/21505594.2024.2329573

**Published:** 2024-03-21

**Authors:** Bruno Montanari Borges, Monique Gama de Santana, Nycolas Willian Preite, Valéria de Lima Kaminski, Gabriel Trentin, Fausto Almeida, Flávio Vieira Loures

**Affiliations:** a Institute of Science and Technology (ICT), Federal University of São Paulo (UNIFESP), São José dos Campos, São Paulo, Brazil; b Department of Biochemistry and Immunology, Ribeirao Preto Medical School, University of São Paulo, São Paulo, Brazil

**Keywords:** Paracoccidioides brasiliensis, extracellular vesicles, proteome, *granuloma*, Paracoccidioidomycosis, dendritic cells, macrophages

## Abstract

Extracellular vesicles (EVs) are membrane-enclosed nanoparticles that transport several biomolecules and are involved in important mechanisms and functions related to the pathophysiology of fungal diseases. EVs from *Paracoccidioides brasiliensis*, the main causative agent of Paracoccidioidomycosis (PCM), modulate the immune response of macrophages. In this study, we assessed the EVs proteome from a virulent *P. brasiliensis* isolated from granulomatous lesions and compared their immunomodulatory ability with EVs isolated from the fungus before the animal passage (control EVs) when challenging macrophages and dendritic cells (DCs). Proteome showed that virulent EVs have a higher abundance of virulence factors such as GP43, protein 14-3-3, GAPDH, as well as virulence factors never described in PCM, such as aspartyl aminopeptidase and a SidJ analogue compared with control EVs. Virulent extracellular vesicles induced higher expression of TLR4 and Dectin-1 than control EVs in macrophages and dendritic cells (DCs). In opposition, a lower TLR2 expression was induced by virulent EVs. Additionally, virulent EVs induced lower expression of CD80, CD86 and TNF-α, but promoted a higher expression of IL-6 and IL-10, suggesting that EVs isolated from virulent *P. brasiliensis*-yeast promote a milder DCs and macrophage maturation. Herein, we showed that EVs from virulent fungi stimulated a higher frequency of Th1/Tc1, Th17, and Treg cells, which gives new insights into fungal extracellular vesicles. Taken together, our results suggest that *P. brasiliensis* utilizes its EVs as virulence bags that manipulate the immune system in its favour, creating a milder immune response and helping with fungal evasion from the immune system.

## Introduction

Paracoccidioidomycosis (PCM) is a granulomatous systemic mycosis restricted to Latin American countries and is one of the most widespread mycoses in Brazil, Venezuela and Colombia. The disease is caused by the thermically dimorphic fungus *Paracoccidioides brasiliensis*, *P. lutzii, P. americana*, *P. venezuelensis* and, *P. restrepiensis* [1,2]. In Brazil, around 5,000 cases of PCM are reported yearly, being responsible for the eighth cause of death among parasitic and infectious diseases and the first among systemic mycotic diseases (1.45 per million inhabitants). The infection occurs after inhalation of conidia or mycelia fragments of the fungus present in the soil, mainly affecting rural workers. After inhalation the fungus remains as a localized benign lesion or evolves as a manifest disease, progressing from a primary lesion or reactivation of a latent pulmonary focus [3–6].

The survival and spread of a pathogen are directly related to the molecules released by them, which can modulate the host’s immune system and be used in pathogen-pathogen communication [7]. Extracellular vesicles (EVs), or exosomes, are plasma membrane-bound nanoparticles that are released by many cell types, ranging from 30 to 1000 nm, although most are in the 100 to 150 nm range. EVs function as a transport vehicle for molecules such as proteins, lipids, and RNAs between the extra and intracellular environment, which is why they are essential for communication between cells [8].

In a pioneer study, it was demonstrated that the EVs isolated from *P. brasiliensis* had highly immunogenic α-Galactosyl epitopes. Those same epitopes were also found within the cell wall and inside large intracellular vacuoles [9]. Later, the same group suggested that *P. brasiliensis* EVs surface is mainly composed of high amounts of terminal mannose oligosaccharides and lower amounts of terminal *N*-acetylglucosamine. It was also demonstrated that those oligosaccharides exposed in the EVs surface were recognized by CD209 from macrophages and DCs [10]. In a proteome study of *P. brasiliensis* EVs, the presence of proteins related to various biological processes, including protein and carbohydrate metabolism, oxidative stress, oxidation, signalling, cell division, and transport, was reported [7].

To access the ability of *P. brasiliensis* EVs to modulate an immune response, macrophages were challenged with EVs, which stimulated the polarization to M1 macrophages. This suggested that the EVs themselves can modulate an inflammatory response, a fact proven by the stimuli of M2 macrophages, which underwent repolarization to M1 after the challenge with EVs [11]. Similarly another group evaluated macrophages challenged with EVs from virulent *P. brasiliensis* and attenuated *P.* *brasiliensis* [12]. The authors observed lower levels of IL-6, TNF-α, and MCP-1 in macrophages challenged with EVs from a virulent fungus when compared to the challenge with EVs from an attenuated fungus. Additionally, the authors showed that the virulent EVs enhance the expression of virulence traits by the attenuated fungus and enhance the fungal burden on infected mice [12]. Furthermore, a study showed that *P. brasiliensis* EVs, in the presence of adjuvants, can be used as immunizers, since they triggered a beneficial effect when administered prior to the infection. Immunization with EVs resulted in increased activation of T lymphocytes, greater migration of natural killer cells to the lungs, with increased expression of pro-inflammatory cytokines and reduced fungal burden [13]. It is worth mentioning that the EVs isolation protocol and the culture medium used in those assays were different from one another and different with the one used in this manuscript, which influence in the EVs cargo [14].

To the best of our knowledge, there are no reports demonstrating that fungal extracellular vesicles can modulate an adaptive immune response. However, in experiments with EVs isolated from *Candida albicans*, it was observed that DCs challenged in vitro with these EVs exhibited an increase in the expression of CD86 and MHC-II, which are crucial molecules for antigen presentation and, consequently, the induction of an adaptive immune response [15]. Although there are no studies that enlighten the ability of fungal EVs to modulate an adaptive immune response, isolated EVs from *Leishmania donovani* have shown the capability to induce a Th1 response in vitro. Interestingly, in the same study, it was observed that EVs from *Leishmania major* triggered a Th2 response in vivo, leading to a more exacerbated infection [16].

The studies with extracellular vesicles of *P. brasiliensis* cited above show the ability of *P. brasiliensis* EVs to modulate an immune response. For this reason, a proteomic analysis of these EVs, as well as an analysis of the immunomodulation of DCs, macrophages, and adaptive response promoted by the EVs, could return interesting results, enabling a greater understanding of the disease and the virulence factors of the fungus that eventually make up the EVs. Herein, we performed a proteomic analysis of the EVs isolated from a virulent *P. brasiliensis* Pb18 strain, that was recently isolated from long-term infections and compared with the EVs proteins of the Pb18 used for infection, before the animal passage. The EVs isolated from the virulent strain exhibited elevated protein levels associated with metabolic processes and oxidative stress. Additionally, we performed immunomodulation assays of DCs and macrophages challenged with the EVs. In response to virulent EVs, antigen-presenting cells had less activation when compared to the stimulus given by the control EVs. Furthermore, the virulent EVs induced a more exacerbated Th1/Tc1 and Th17 immune responses, concomitant with a higher frequency of Treg, showing for the first time the ability of fungal EVs to modulate a cellular adaptive response.

## Material and methods

### Ethics statement

All the experiments were performed in strict accordance with the Brazilian Federal Law 11,794 establishing procedures for the scientific use of animals, and the State Law establishing the Animal Protection Code of the State of São Paulo. All efforts were made to minimize animal suffering. The procedures were approved by the Ethics Committee on Animal Experiments of the Federal University of São Paulo (N° 9596270919).

### Animals

Throughout this study, C57BL/6 WT male mice aged between 8 and 12 weeks, acquired from the Center for the Development of Experimental Models for Biology and Medicine (CEDEME-UNIFESP), and bred as mice free from specific pathogens in the animal facility of the Institute of Science and Technology at UNIFESP, were utilized. All procedures with mice were approved by the Ethics Committee on the Use of Animals of UNIFESP (Nº 9596270919).

#### Paracoccidioides brasiliensis

The strain Pb18 of the *P. brasiliensis* fungus was utilized and maintained through sub-cultivation on a semi-solid Fava Netto medium at a temperature of 37°C [17]. For mice infection, the fungal sample was collected and rinsed with phosphate-buffered saline (PBS, pH 7.4). The yeast count was measured utilizing a haemocytometer, and their vitality was ascertained with the Janus-Green vital stain. All steps were conducted using a fungal mixture with a viability exceeding 95%.

## Mice infection

Groups of C57BL/6 WT mice were anesthetized with intraperitoneal (ip.) injection of ketamine (90 mg/kg) and xylazine (10 mg/kg) and submitted to intratracheal (it.) infection as previously described [18]. In summary, the mice were infected with 1 × 10^6^ *P. brasiliensis* yeasts, suspended in 50 μL of PBS, through a surgical (it.) inoculation that allowed direct delivery of the yeasts into the lungs.

### Granulomatous lesions extraction and virulent yeast recovery

A previous report by our group demonstrated that culturing the yeast present in the granulomatous lesions enhances the virulence of the Pb18 yeast [19]. Accordingly, to obtain a Pb18 with higher virulence, after eight and 12 weeks of infection, 3 independent groups with 2–4 animals were euthanized, the lungs were removed, and the granulomatous lesions were separated from the apparently healthy lung regions with the help of a scalpel as previously described [19]. The lesions were macerated with a Doucer, centrifuged (1,200 × g, 10 minutes), and then the pellet was resuspended in PBS. The recovered yeasts were cultivated in BHI medium (Brain Heart Infusion, Merck) for 5 days before cultivation in Fava Netto medium for 7 days (recovered yeast). The cultivation of the yeasts in BHI before cultivation in Fava Netto medium is necessary because there is growth factor in the BHI composition, which is essential for fungal growth after recovery from the lesions, as previously described [20].

### Isolation of extracellular vesicles

The EVs were extracted from virulent yeasts (vEVs; yeasts obtained from 8 and 12 weeks of infection) and control (cEVs, yeasts used for mice infection) according to [21]. Briefly, yeasts of *P. brasiliensis* were recovered from Fava Netto medium with the help of a loop and placed in a 50 mL tube containing 30 mL of sterile PBS. The suspension was centrifuged at 4,000 × g for 15 minutes at 4^º^C and the supernatant was transferred to a new 50 mL tube for centrifugation at 15,000 × g for 25 minutes at 4^º^C. The supernatant was recovered and filtered using a 0.45 μm pore syringe filter before centrifuging at 100,000 × g, 4°C for 1 hour. The supernatant was discarded, and the pellet was resuspended in 500 μL of sterile PBS and filtered through a 0.45 μm filter. To optimize vEVs concentration, a pool of fungi originated from the same infection group was created.

### EVs quantification by nanoparticle tracking analysis (NTA)

Nanoparticle tracking analysis was performed using the Nanosight NS300 appliance (Malvern Instruments, Malvern, UK) with NTA 3.0 software. The parameters used were those recommended by the manufacturer’s manual. The camera level was increased to a level > 14, at which all particles were visible. The threshold was determined to capture as many particles as possible within an optimal range of 20 to 100 particles per frame.

### EVs protein extraction and trypsin digestion

The protein content of the EVs was extracted using Tris-SDS buffer, as described by [22]. Briefly, SDS-Tris buffer [2% sodium dodecyl sulphate (SDS) (Sigma Aldrich), 20 mM Tris.HCl pH 8 (Sigma Aldrich)] was added to the EVs solution in a 1:1 ratio. Then, the solution was homogenized and heated at 95°C for 5 minutes and then placed in an ice-cold sonication bath. Six sonication cycles were performed for 30 seconds with 30-second intervals on ice to reduce overheating. The method of in-solution trypsin digestion, as described by [23] with some modifications [23], was performed. To summarize, a 6 M guanidine hydrochloride (GuHCl) solution was added to a sample containing 100 μg of protein from each cell lysate, resulting in a final concentration of 3 M GuHCl. Next, 5 mM dithiothreitol (DTT) was added (final concentration), and the mixture was incubated at 37^º^C for 1 h. Iodoacetamide (IAA) was then added to a final concentration of 15 mM, and the samples were incubated in the dark at room temperature for 30 minutes. To quench the IAA excess, 15 mM DTT was added, followed by a 20-minute incubation at room temperature. Sample cleanup was carried out by adding ice-cold acetone (8 volumes) and methanol (1 volume), followed by a 3-hour incubation at −80^º^C. After centrifugation at 14,000 × g for 10 minutes, protein pellets were washed twice with ice-cold methanol. The pellets were then resolubilized with a NaOH solution (10 mM final concentration) and supplemented with HEPES buffer (50 mM final concentration, pH 7.5) to a final volume of 100 μL. Trypsin (Proteomics grade; Sigma, USA) was added at a ratio of 1:100 (enzyme/substrate), and the protein samples were incubated at 37^º^C for 18 h.

The resulting tryptic peptides were desalted using C-18 cartridges SPE Extraction disks (3 M EmporeTM), resuspended in 50 μL of 0.1% formic acid, quantified using the Pierce™ Quantitative Colorimetric Peptide Assay (Thermo Scientific), and stored at −80^º^C.

### LC-MS/MS analysis and protein identification

An aliquot containing 1 µg of peptide mixture was analysed utilizing an LTQ Orbitrap Velos (Thermo Fisher Scientific) mass spectrometer, combined with nanoflow liquid chromatography via an EASY-nLC system (Proxeon Biosystems) featuring a Proxeon nanoelectrospray ion source. Subsequently, peptides were separated within a gradient of 2–90% acetonitrile in 0.1% formic acid, utilizing a PicoFrit analytical column (20 cm × ID75, 5 µm particle size, New Objective) at a flow rate of 300 nL/min over a span of 212 min, in which a 35% acetonitrile gradient was achieved within 175 min. The nanoelectrospray voltage was established at 2.2 kV, and the source temperature was set to 275^º^C. The instrument methodologies for the LTQ Orbitrap Velos were configured in DDA mode. Following accumulation to a target value of 1 × 10^6^, full scan MS spectra (m/z 300–1600) were acquired in the Orbitrap analyser. The Orbitrap resolution was set to *r* = 60,000, and the top 20 most intense peptide ions with charge states ≥ 2 were consecutively isolated to a target value of 5000 and subjected to fragmentation via CID (collision-induced dissociation) in the high-pressure linear ion trap, utilizing a normalized collision energy of 35%. Dynamic exclusion was activated, employing an exclusion size list of 500 peptides, with an exclusion duration of 60 s and a repetition count of 1. An activation Q of 0.25 and an activation time of 10 ms were employed. Mass spectrometric (RAW) data were analysed with MaxQuant software (version 2.0.3.0). A False Discovery Rate (FDR) of 1% was required for both protein and peptide-to-spectrum match identifications. Raw data were searched against a target database restricted to the taxonomy “*Paracoccidioides brasiliensis”* (UniProt/Proteomes – UP000001628; 8,399 entries). This database was also combined with the sequences of 245 common contaminants and concatenated with the reversed versions of all sequences. Enzyme specificity was set to trypsin and up to two missed cleavages were allowed; cysteine carbamidomethylating was selected as fixed modification whereas methionine oxidation, glutamine/asparagine deamidation and protein N-terminal acetylation were selected as variable modifications. Peptide identification was based on a search with an initial mass deviation of the precursor ion of 4.5 ppm and the fragment mass tolerance was set to 20 ppm. Label-free quantitation was performed using the MaxLFQ algorithm [24,25] with the “re-quantify” function of MaxQuant software enabled. As observed in complex proteomes such as those of vertebrates, peptides can be shared between homologous proteins or splice variants, leading to “protein groups.” For each protein group in the MaxQuant’s “proteinGroups.txt” file, the first protein entry was selected as representative.

### Differentially abundance of proteins and functional analysis

For the proteomics data, the protein intensity values were subjected to log2 transformation and quantile normalization using the “preprocessCore” library in the R scripting and statistical environment [26,27]. Subsequently, statistical analyses were conducted using the “limma” package in R/Bioconductor, employing eBayes [28–30]. Differentially abundant proteins were identified based on an adjusted *p*-value <0.05 and a fold change cut-off of |fold change| > 1, with *p*-values adjusted for multiple testing using the Benjamini-Hochberg method. For function prediction, Kyoto Encyclopedia of Genes and Genomes (KEGG) and Gene Ontology (GO) annotations were taken [31]; [32,33,34,35]. The *P. brasiliensis* Gene Ontology functions were taken from FungiDB.

### Generation of bone marrow dendritic cells and macrophages

C57BL/6 WT mice had their femurs and tibias collected; the muscles were carefully removed with a scalpel under aseptic conditions. Then Roswell Park Memorial Institute (RPMI) 1640 medium (Sigma Aldrich) supplemented with 3% foetal bovine serum (LGC biotecnologia, Cotia, São Paulo) and 1% streptomycin/penicillin (Sigma Aldrich) was used to wash the bone marrow and collect the cells. The cells were then centrifuged at 300 × g, 10 minutes, 4°C. The pellet was resuspended in 4 mL of red blood cell (RBC) lysis buffer (BioLegend), for 4 minutes. Then, 20 mL of RPMI supplemented with 3% foetal bovine serum (FBS) was added to neutralize the RBC lysis buffer. The cells were centrifuged again and the precipitated cells were resuspended in RPMI supplemented with 10% FBS and cultured in 75 cm^3^ bottles containing up to 1.5 × 10^6^ cells in the presence of 20 ng/mL rmGM-CSF (recombinant mouse granulocyte and macrophage colony-stimulating factor). Cultivation was carried out for 5 days at 37ºC in a 5% CO_2_ chamber, with a change of RPMI containing rmGM-CSF on the second and fourth day of cultivation. On the fifth day of culture, the suspended were recovered and the adhered were obtained with the help of a cell scraper. Then, the cells were centrifuged and resuspended in RPMI medium supplemented with 10% FBS.

### Characterization of macrophages and dendritic cells challenged with EVs

The cells (1 × 10^6^), plated in 48-well flat-bottom plates, were challenged with EVs from control or virulent yeasts (fungus pool obtained from 8 weeks of infection) at concentrations of 10^7^, 10^8^, and 10^9^ EVs/mL. As a positive control, lipopolysaccharide (LPS) was used at a concentration of 1 µg/mL per well. The challenged cells were incubated at 37^º^C, 5% CO_2_ for 48 hours, and then, the supernatants were recovered for enzyme-linked immunosorbent assay (ELISA). The cells were characterized by flow cytometry. The Fc receptors were blocked with unlabelled anti-CD16/32 (eBioscience) according to the manufacturer’s instructions and stained for 30 min at 4°C with fluorophore-conjugated antibodies. After exclusion of dead cells using Live/Dead BV510 antibody (BD Biosciences), macrophages were defined as CD11b^+^CD11c^+^F4/80^+^, while DCs were defined as CD11b^+^CD11c^+^F4/80^−^ and the results expressed as the percentage of these cells expressing CD40, CD80, CD86, MHCII, TLR2, TLR4, Dectin-1, IL-6 and IL-10. Cell populations were acquired using the FACS Lyric equipment (BD Biosciences), with the acquisition of 60,000 events per sample. Analyses were performed using FlowJo v. 6.10. The gating strategy is shown in Supplementary Figure S1.

### Cytokines and chemokines analysis

The levels of MCP-1, GM-CSF, TNF-α, and IL-1β were determined with the supernatants collected from the culture of cells challenged with the control and virulent EVs, using capture ELISA. The kits used were ELISA MAX™ Deluxe Set Mouse MCP-1 kits (BioLegend), ELISA MAX™ Deluxe Set Mouse GM-CSF (BioLegend), ELISA MAX™ Deluxe Set Mouse TNF-α (BioLegend), and ELISA MAX™ Deluxe Set Mouse IL-1β (BioLegend). Assays were conducted according to the protocols for each kit. Plates were read using a spectrophotometric plate reader (Synergy H1, Biotek).

### Evaluation of the adaptive response promoted by the EVs

After the challenge of the innate cells with the extracellular vesicles, a co-culture was performed with lymphocytes obtained from the spleens of naïve mice in order to observe the adaptive response patterns developed by the lymphocytes. Lymphocyte populations were separated from splenocytes using the Pan T Cell Isolation Kit II (Milteniy Biotec). Antigen-presenting cells (APCs) challenged with EVs were placed in co-culture with lymphocytes in a 96-well round-bottom plate (1:10, APC-lymphocytes ratio). Cultivation was made for 5 days, with RPMI medium, supplemented with 10% FBS, change on the second and fourth day. Cells were characterized by multiparametric flow cytometry. A minimum of 50,000 events were acquired on the FACSLyric flow cytometer (BD Biosciences) using FlowJo software (Tree Star) for analyses. Cell populations were defined as: regulatory T lymphocytes (CD4^+^ CD25^+^ FoxP3^+^), Th1 lymphocytes (CD4^+^ IFNγ^+^), Th2 (CD4^+^ IL-4^+^), Th17 (CD4^+^ IL-17^+^), Tc1 (CD8^+^ IFNγ^+^), Tc2 (CD8^+^ IL-4^+^), Tc17 (CD8^+^ IL-17^+^). The gating strategy is represented in Supplementary Figure S2.

### Statistical analysis

For statistical analysis, mean ± standard deviation/standard error was compared. For comparison between two groups, an unpaired Student’s t-test was performed, while for multiple analyses a one-way ANOVA was performed, with Bonferroni correction. The significance level was defined as *p* < 0.05.

## Results

### Virulent EVs contain higher abundance of virulence factors and energy metabolism proteins than cEvs

In a previous work by our group, we demonstrated that the yeasts present in the granulomatous lesions reduce their metabolic activity to avoid energy loss and survive inside the host. Nevertheless, after culturing granulomatous yeasts, their metabolic activity and virulence increased when compared to control yeasts, those used to inoculate the mice [19]. In this present work, we analysed the EVs produced by those virulent fungi (vEVs) and compared them to the EVs produced by the fungi used in the infection (cEVs).

**Figure 1.**
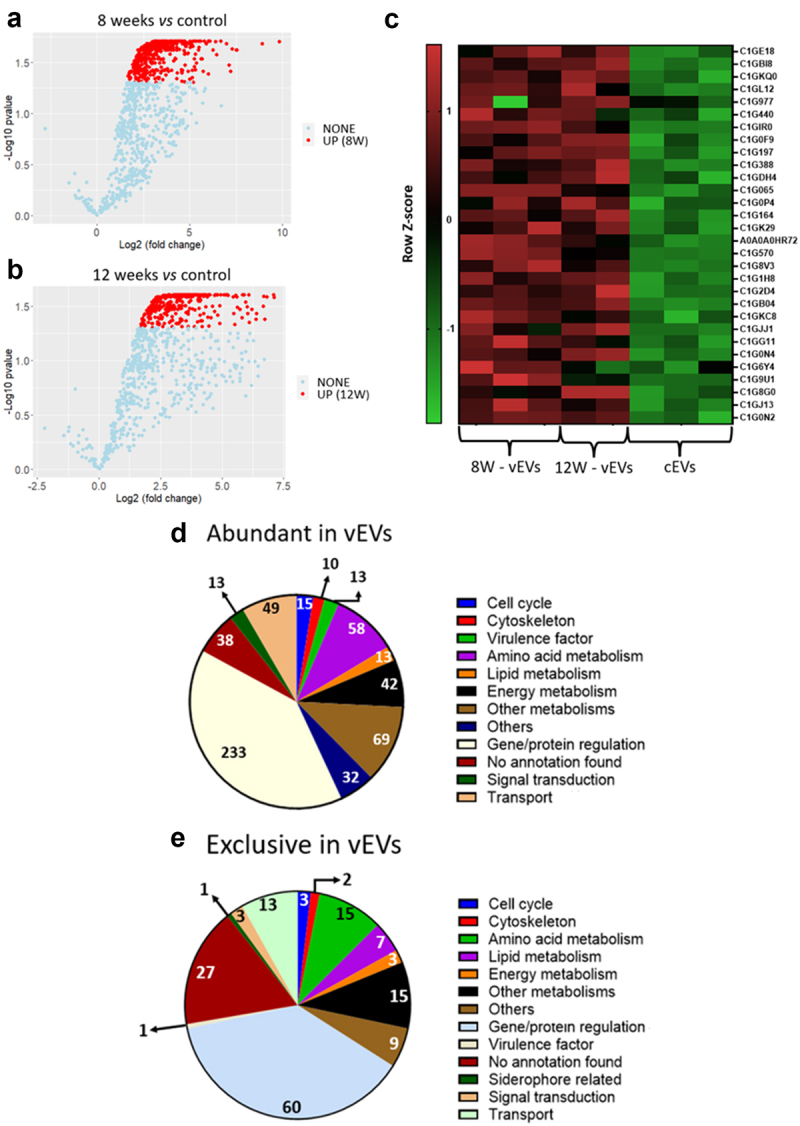
vEVs contain high abundance of virulence factors and energy metabolism proteins. Granulomatous lesions were extracted from two to four mice from three independent infections at eight weeks and 12 weeks after infection with 1 × 10^6^ *P. brasiliensis* yeasts. After culturing the fungus extracted from the lesions, EVs were isolated, and their protein content was extracted and digested with trypsin and resolubilized in 0.1% formic acid. Peptides were analysed by LC-MS/MS and proteins were identified by MaxQuant software. Proteins containing more than two unique peptides had their intensity normalized by log2 followed by quantile normalization within each experimental group. Then, a differentially abundant protein analysis was made using limma and a functional analysis was carried out. To illustrate the differentially abundant proteins of the EVs, a volcano plot of the 8-weeks infection (8W – vEVs) group in relation to the control EVs (a) and a volcano plot of the 12-weeks infection group (12W – vEVs) in relation to the control EVs (b) were made. Additionally, a heatmap was created with selected proteins (c). The proteins in red represent a higher abundance. The functions of the more abundant proteins (d), and the protein found exclusively in the vEVs proteins (e) were taken using GO, KEGG, and FungiDB.

The analysis of differentially abundant proteins shows a high number of abundant proteins in vEVs when compared to cEVs. vEVs isolated from 8-week granulomatous lesions yeasts contained 463 proteins and none were repressed in comparison to the cEVs. Similarly, when comparing the proteins within vEVs from yeasts recovered from 12-week granulomatous lesions, 372 proteins were found to be more abundant (Figure 1(a–c)). Conversely, no differences in protein abundance were observed when comparing the proteins extracted from both vEVs groups.

The abundant proteins identified in vEVs were associated with functions related to virulence factors, energy metabolism, lipid and amino acid metabolism, gene/protein regulation, among others. This indicates that the inter-species communication mediated by the EVs isolated from granulomatous yeasts demonstrates a profile of inducing virulence mechanisms and metabolic responses (Figure 1(d), Table 1 and Supplementary Table S1). Among the virulence factors found we can mention the thioredoxin (C1GE18), which is associated with oxidative stress response; glyceraldehyde-3-phosphate dehydrogenase (GAPDH; C1G5F), protein 14-3-3; and glucan 1–3-beta glucosidase (GP43; C1GK29), that help in pathogen adhesion. It is important to emphasize that protein 14-3-3 canonical function is related to cell cycle. Additionally, proteins related to the cell wall composition, which are also virulence factors, were found in greater abundance in vEVs compared to cEVs, including 1,3-beta-glucan synthase (A0A0A0HS55) [36]. The presence of virulence factors with higher abundance within the vEVs when compared to cEVs can indicate that some virulence mechanisms might be modulated by the EVs produced by the fungus.

**Table 1.** Selected proteins abundant in vEvs.

Acession number	Protein	Expression status	Log (Fold change)	Adjusted p-Value
**Virulence factor**				
C1GE18	Thioredoxin	UP(D)	4,131451	0,000105
C1GB04	14-3-3 protein epsilon	UP(D)	2,719102	4,88E–05
C1GK29	Glucan 1,3-beta-glucosidase	UP(D)	2,596273	0,000332
A0A0A0HS55	1,3-beta-glucan synthase	UP(D)	2,298877	0,000759
C1G5F6	Glyceraldehyde-3-phosphate dehydrogenase	UP(D)	2,169445	0,000128
C1GBT0	Aldehyde dehydrogenase	UP(D)	1,852013	0,0002
**Gene/protein regulation**				
C1GGX0	Elongator complex protein 2	UP(D)	5,5947	3,89E–05
C1G8V3	tyrosyl-tRNA synthetase [EC:6.1.1.1]	UP(D)	5,396857	5,91E–05
C1GDQ7	Scavenger mRNA decapping enzyme	UP(D)	5,295471	0,000405
C1G0P0	Hsp7-like protein	UP(D)	4,759801	5,66E–05
C1GL49	replication factor A1	UP(D)	3,927564	8,23E–05
C1G695	ATP-dependent DNA helicase II subunit 1	UP(D)	3,817383	4,39E–05
C1G435	GPI mannosyltransferase 2	UP(D)	3,616139	3,44E–05
**Energy metabolism**				
C1GI92	Cytochrome c	UP (8S)	8,920571	0,020855
C1GL50	NADPH2:quinone reductase	UP(D)	6,671233	5,33E–05
C1GBI8	Ribose-5-phosphate isomerase	UP(D)	5,144232	3,44E–05
C1G2P3	Enoyl-CoA hydratase	UP(D)	4,382212	3,44E–05
C1G977	Hexokinase	UP (12S)	4,342947	0,032162
C1GCI8	2-methylcitrate synthase, mitochondrial	UP(D)	3,832717	4,39E–05
C1GL12	Glycogen debranching enzyme	UP(D)	2,979608	0,000124
**Amino acid metabolism**				
C1G3Q0	Methylthioribulose-1-phosphate dehydratase	UP(D)	4,193475	5,07E–05
C1GF86	Cys-Gly metallodipeptidase DUG1 [EC:3.4.13.-]	UP(D)	4,12676	0,000208
C1G6H2	threonine synthase [EC:4.2.3.1]	UP(D)	3,898842	8,58E–05
C1G6P2	2,4-dihydroxyhept-2-ene-1,7-dioic acid aldolase	UP(D)	3,82139	4,39E–05
C1G0F9	kynureninase [EC:3.7.1.3]	UP(D)	3,62137	9,06E–05
C1FYE6	Glutaminase A	UP(D)	3,433912	0,000121
**Lipid metabolism**				
C1GDH4	leukotriene-A4 hydrolase [EC:3.3.2.6]	UP(D)	4,263176	0,000274
C1GMW7	Sphingolipid C9-methyltransferase A	UP (8S)	3,572532	0,037729
C1G0P4	long-chain acyl-CoA synthetase [EC:6.2.1.3]	UP (12S)	3,064416	0,032289
C1GBJ2	ATP citrate synthase	UP(D)	2,453147	0,000166
**Other metabolisms**				
C1G421	acetyl-CoA C-acetyltransferase	UP(D)	6,076812	4,39E–05
C1G3Y2	Alpha-1,6-mannosyltransferase subunit	UP (8S)	4,92358	0,038532
C1G7A4	Alpha-galactosidase	UP(D)	3,633348	0,000115
**Cell cycle**				
C1FYS5	Microtubule-associated protein RP/EB family member 3	UP(D)	4,63316	6,72E–05
C1G5N0	Casein kinase II subunit alpha	UP(D)	3,547176	4,39E–05
A0A0A0HUN4	Mitotic checkpoint protein BUB3	UP(D)	3,294945	6,61E–05
**Signal transduction**				
C1G501	Calmodulin	UP (8S)	7,115894	0,033522
C1GG36	Calcineurin subunit B	UP(D)	2,285403	9,06E–05
**Others**				
C1GA81	Aspartyl aminopeptidase	UP(D)	8,596116	9,61E–05
C1GGK1	Chitin synthase	UP(D)	3,727287	4,11E–05

Granulomatous lesions were extracted from two to four mice from three independent infections at eight weeks and 12 weeks after being infected with 1 × 10^6^ *P.* *brasiliensis* yeasts. After culturing the fungus extracted from the lesions, EVs were isolated, and their protein content was extracted and digested with trypsin and resolubilized in 0.1% formic acid. Peptides were analysed by LC-MS/MS and proteins identified by MaxQuant software. Proteins containing more than two unique peptides had their intensity normalized by log2 followed by quantile normalization within each experimental group. The limma package was used for differentially abundant proteins analysis and GO, KEGG and FungiDB were used for functional analysis. The table shows the differentially abundant proteins with the values of log (Fold change) and adjusted *p*-value. The expression status column represents the more abundant proteins for the eight-week infection group EVs (8), the twelve-week infection group EVs (12), and the proteins that were differentially expressed in both infection groups were combined and reanalysed, resulting in an expression status designation for the disease (D).

Several proteins with gene/protein regulation functions were found in greater abundance in vEVs, like the Elongator complex protein 2 (C1GGX0), tyrosyl-tRNA synthetase (C1G8V3), replication factor A1 (C1GL49) and GPI mannosyltransferase 2 (C1G435) when compared to the protein content of cEVs. Additionally, a high number of proteins related to energy metabolism, such as cytochrome C (C1GBI8), NADPH2:quinone reductase (C1GL50), hexokinase (C1G977), and mitochondrial 2-methylcitrate synthase (C1GCI8) were also found in the proteome of vEVs. We also detected a higher abundance of proteins in the vEVs that participate in amino acid metabolism (threonine synthase (C1G6H2)), lipid metabolism (leukotriene-A4 hydrolase (C1GDH4)) and other metabolisms (alpha-galactosidase (C1G7A4)). Some proteins related to cell cycle and signal transduction proteins were also found with high abundance in vEVs, such as casein kinase II subunit alpha (C1G5N0), mitotic checkpoint protein BUB3 (A0A0A0HUN4), calmodulin (C1G501) and calcineurin subunit B (C1GG36). The aspartyl aminopeptidase protein (C1GA81), which has already been described as a possible virulence factor for *Cryptococcus neoformans*, was also in higher levels in vEVs in comparison to cEVs [37].

Likewise, proteins found exclusively in the vEVs (Figure 1(e), Table 2 and Supplementary Table S2) and proteins that were absent in those EVs (Supplementary Figure S1 and Supplementary Table S3) were also analysed. The exclusive proteins to vEVs primarily serve gene/protein regulation functions, followed by general metabolism and transport proteins. Curiously, the protein C1G0T3 (glycosyl transferase CAP10 domain-containing protein), which is a homologue of a protein responsible for the formation of capsules in *C. neoformans* [38] was found only in the vEVs. Furthermore, the protein C1G110 (fusarinin C esterase; SidJ), which is homologous to SidJ from *Aspergillus fumigatus* and has the function of cleaving the iron-chelated fusarinin C bond, releasing iron into the intracellular environment, was also found exclusively in vEVs [39]. Interestingly, most of the proteins that were absent in the virulent EVs, and therefore exclusive to the control group, are associated with gene/protein regulation functions, and two proteins that have already been described as virulence factors were also absent within the vEVs.

**Table 2.** Selected proteins exclusive in vEvs.

Acession number	Protein
**Virulence factor**	
C1G0T3	Glycosyl transferase CAP10 domain-containing protein
**Gene/protein regulation**	
C1FZJ4	Transcription elongation factor S-II
C1G573	rRNA biogenesis protein RRP5
C1G582	DnaJ domain-containing protein
C1GAR5	Alpha 1,2-mannosyltransferase
C1GBK4	Ubiquitin-activating enzyme E1-like
**Energy metabolism**	
C1FZL2	Succinate dehydrogenase [ubiquinone] flavoprotein subunit, mitochondrial[…]
C1GAG2	NADH dehydrogenase [ubiquinone] 1 alpha subcomplex subunit 13
**Amino acid metabolism**	
A0A0A0HWJ2	Amidase 1
C1G025	Aromatic-L-amino-acid decarboxylase
C1GLQ9	Ornithine carbamoyltransferase, mitochondrial
**Lipid metabolism**	
C1GFA5	Phospholipase D
C1GFW9	Palmitoyl-protein thioesterase 1
**Other metabolisms**	
A0A0A0HUH4	Thiamine diphosphokinase
C1GB66	Allantoate amidohydrolase
C1GBL6	Chitin deacetylase
C1GFB5	Aldose 1-epimerase
**Siderophore related**	
C1G110	Fusarinine C esterase sidJ

Granulomatous lesions were extracted from two to four mice from three independent infections at eight weeks and 12 weeks after being infected with 1 × 10^6^ *P. brasiliensis* yeasts. After culturing the fungus extracted from the lesions, EVs were isolated, and their protein content was extracted and digested with trypsin and resolubilized in 0.1% formic acid. Peptides were analysed by LC-MS/MS and proteins identified by MaxQuant software. Proteins containing more than two unique peptides had their intensity normalized by log2 followed by quantile normalization within each experimental group.

### vEVs induce high expression of TLR4 and Dectin-1 but low expression of TLR2 in DCs and macrophages

**Figure 2.**
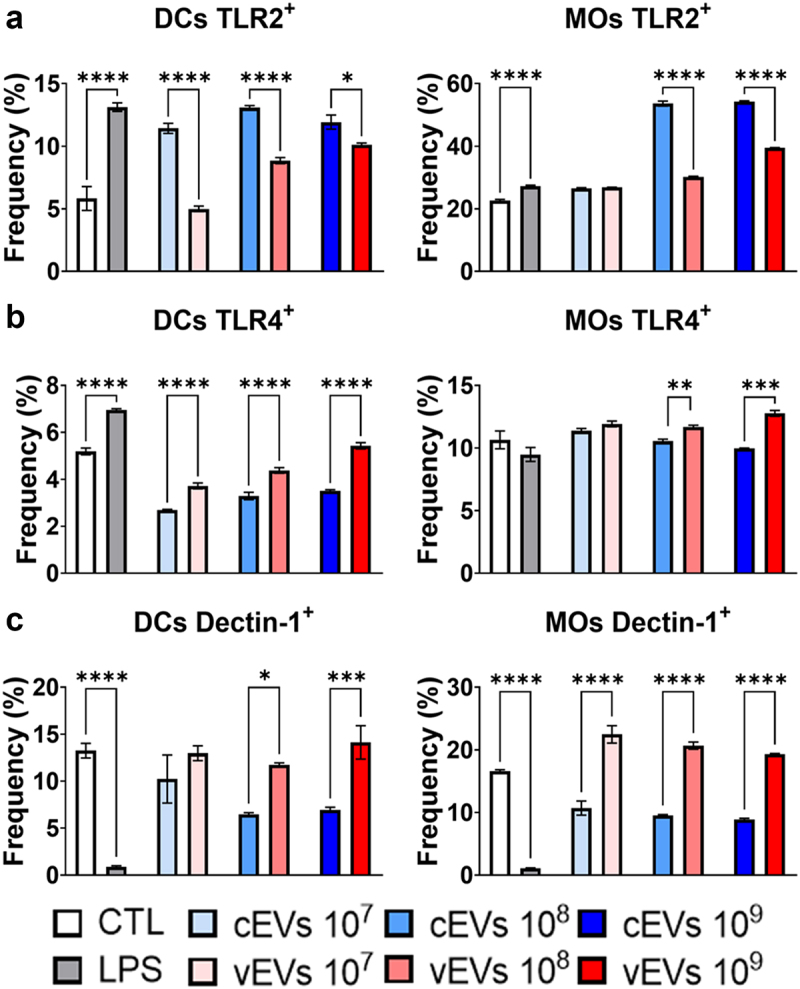
vEVs induce high expression of TLR4 and dectin-1 but low expression of TLR2 in DCs and macrophages. Antigen-presenting cells obtained from the bone marrow cells of C57BL/6 mice, which were differentiated with GM-CSF (20 ng/mL), were challenged with different concentrations of *P. brasiliensis* control and virulent EVs (10^7^, 10^8^, and 10^9^ EVs/mL) for 48 hours. The cells were then recovered and labelled with fluorochrome-conjugated antibodies. Flow cytometry using FACSLyric and FlowJo software was used to analyse the frequency of DCs (CD11b^+^CD11c^+^F4/80^−^; left) and macrophages (CD11b^+^CD11c^+^F4/80^+^; right) expressing TLR2 (a), TLR4 (b), and dectin-1 (c). The bars represent means ± standard error of triplicates per group (*p < 0.05; **p < 0.01; ***p < 0.001; ****p < 0.0001). As a negative control (CTL), cells received only RPMI medium, while LPS (1 µg/mL) was used as positive control.

To comprehend the role of *P. brasiliensis* EVs in modulating macrophages and DCs an analysis of the innate immunity receptors TLR2, TLR4, and Dectin-1 was performed. The TLR2 expression (Figure 2(a)) was reduced in samples challenged with vEVs when compared to the cells challenged with cEVs. Nevertheless, the TLR4 expression (Figure 2(b)) was increased in samples challenged with vEVs. Likewise, Dectin-1 expression was also higher in cells challenged with the vEVs when compared to the stimuli given by cEVs (Figure 2(c)).

### vEVs induce low expression of costimulatory molecules in DCs and macrophages

**Figure 3.**
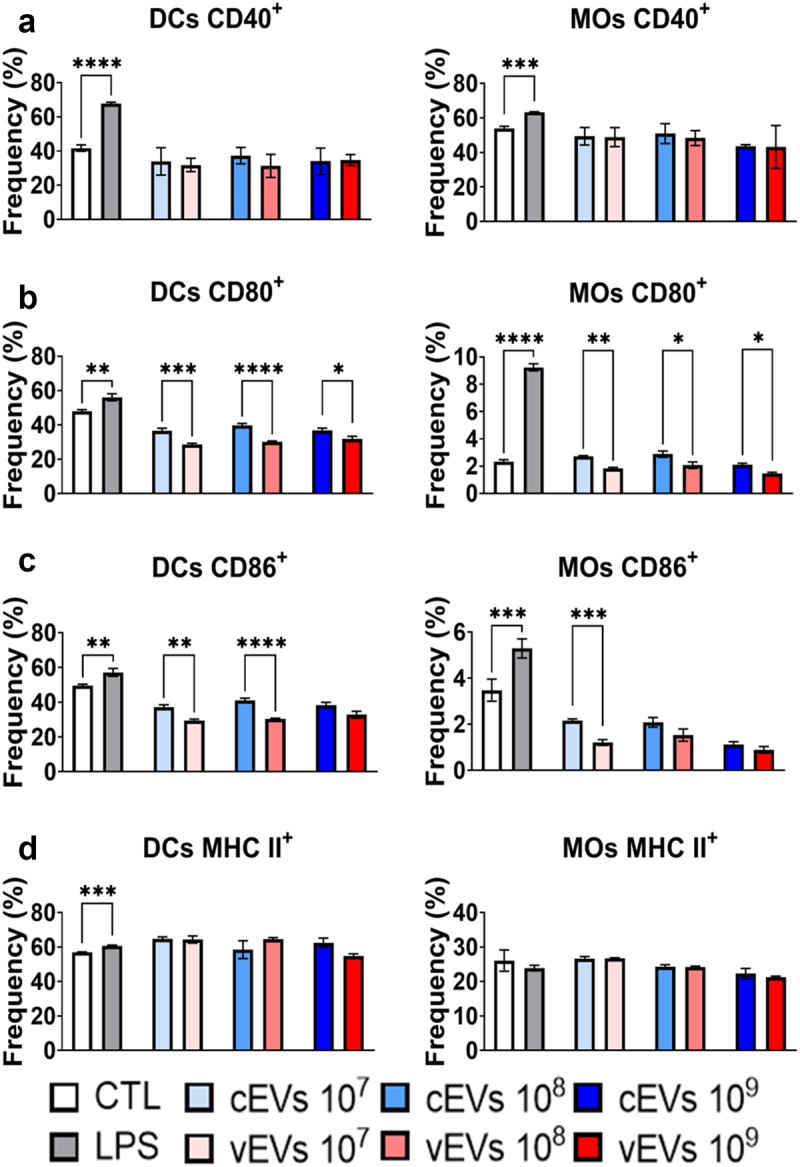
vEVs promote compromised maturation of DCs and macrophages than cEvs. Antigen-presenting cells obtained from the bone marrow cells of C57BL/6 mice, which were differentiated with GM-CSF (20 ng/mL), were challenged with different concentrations of *P. brasiliensis* control and virulent EVs (10^7^, 10^8^, and 10^9^ EVs/mL) for 48 hours. The cells were then recovered and labelled with fluorochrome-conjugated antibodies. Flow cytometry using FACSLyric and FlowJo software was used to analyse the frequency of DCs (CD11b^+^CD11c^+^F4/80^−^; left) and macrophages (CD11b^+^CD11c^+^F4/80^+^; right) expressing CD40 (a), CD80 (b), CD86 (c), and MHC II (d). The bars represent means ± standard error of triplicates per group (*p < 0.05; **p < 0.01; ***p < 0.001; ****p < 0.0001). As a negative control (CTL), cells received only RPMI medium, while LPS (1 µg/mL) was used as positive control.

Another assessment made on the modulation of cells was regarding the molecules that characterize the activation profile of these cells. The costimulatory molecule CD40 (Figure 3(a)) exhibited no significant difference in its expression in cells challenged with vEVs compared to those challenged with cEVs. However, a reduction in CD80 expression was observed (Figure 3(b)), along with CD86 (Figure 3(c)), in cells challenged with vEVs. This suggests a milder activation of cells challenged with vEVs. Moreover, the expression of the antigen-presenting molecule MHC II (Figure 3(d)) demonstrated no distinguished difference between the groups of macrophages and dendritic cells challenged with cEVs and vEVs.

### Cells challenged with vEVs have impaired production of TNF-α, MCP-1, and GM-CSF

**Figure 4.**
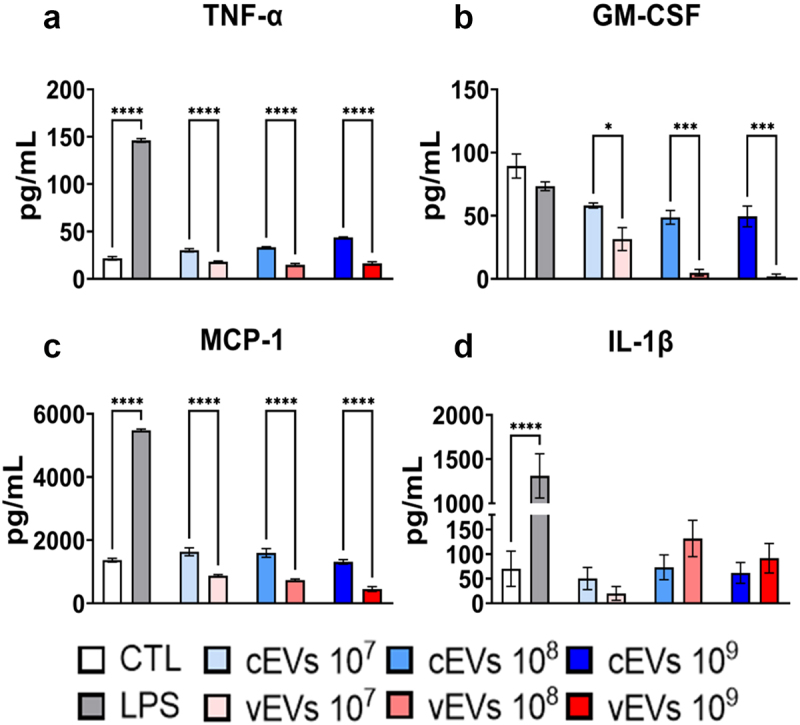
vEVs enhance the expression of intracellular IL-6 and IL-10 when compared to expression stimulated by cEVs. Antigen-presenting cells obtained from the bone marrow of C57BL/6 mice, which were differentiated with GM-CSF (20 ng/mL), were challenged with different concentrations of *P. brasiliensis* control and virulent EVs (10^7^, 10^8^, and 10^9^ EVs/mL) for 48 hours. The cells were then recovered and labelled with fluorochrome-conjugated antibodies. Flow cytometry using FACSLyric and FlowJo software was used to analyse the frequency of DCs (CD11b^+^CD11c^+^F4/80^−^; left) and macrophages (CD11b^+^CD11c^+^F4/80^+^; right) expressing IL-6 (a), IL-10 (b), and TNF-α (c). The bars represent means ± standard error of triplicates per group (*p < 0.05; **p < 0.01; ***p < 0.001; ****p < 0.0001). As a negative control (CTL), cells received only RPMI medium, while LPS (1 µg/mL) was used as a positive control.

From the supernatant of the culture of cells challenged with the EVs, an ELISA assay was performed, aiming to evaluate the profile of cytokines secreted by these innate cells. The cytokines TNF-α and GM-CSF, and the chemokine MCP-1 were found in lower levels in samples challenged with vEVs when compared to the stimulus given by cEVs (Figure 4(a–c)). This indicates that EVs from fungi obtained from lesions induce impaired secretion of important cytokines in the antifungal response given by cells. However, no differences were found in the levels of IL-1β secreted by cells challenged by either cEVs or vEVs (Figure 4(d)).

### vEVs enhance the expression of intracellular IL-6 and IL-10 when compared to expression stimulated by cEVs

**Figure 5.**
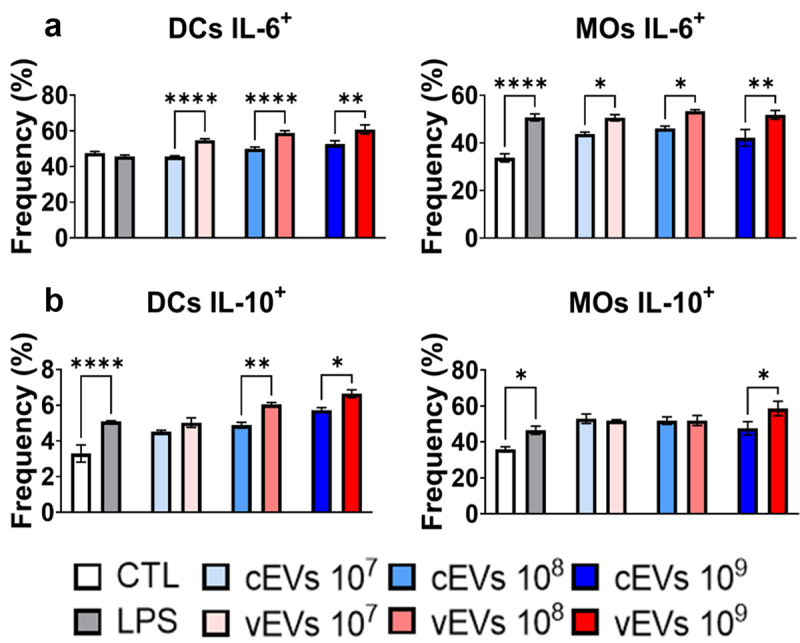
Antigen-presenting cells secrete lower levels of MCP-1, GM-CSF, and TNF-α when stimulated with vEVs than cEVs. Antigen-presenting cells obtained from the bone marrow of C57BL/6 mice, which were differentiated with GM-CSF (20 ng/mL), were challenged with different concentrations of *P. brasiliensis* control and virulent EVs (10^7^, 10^8^, and 10^9^ EVs/mL) for 48 hours. The supernatant was collected and the levels of MCP-1 (a), GM-CSF (b), TNF-α (c), and IL-1β (d) were dosed by ELISA. The bars represent means ± standard error of triplicates per group (*p < 0.05; ***p < 0.001; ****p < 0.0001). As a negative control (CTL), cells received only RPMI medium, while LPS (1 µg/mL) was used as positive control.

For the cytokines that were not detected through ELISA, a flow cytometry approach was employed. This allowed the analysis of intracellular IL-6 and IL-10 production specifically by DCs and macrophages. For both DCs and macrophages that were challenged with the vEVs, an increase in the cytokine IL-6 was seen when compared to the cells stimulated with cEVs (Figure 5(a)). Additionally, an increase in IL-10 expression was also observed in cells challenged with vEVs, but only with the highest concentration of EVs used (10^8^ and 10^9^ EV/mL) (Figure 5(b)). This evidence suggests a dual role played by DCs and macrophages in both inflammatory and anti-inflammatory immune responses.

### DCs primed with vEVs induce a higher frequency of Th1/Th17 cells compared to DCs primed with cEVs

An adaptive immune response profile promoted by fungal EVs has not yet been described, but it is known that EVs isolated from *L. donovani* were capable of inducing a Th1 response in vitro. Interestingly, in the same work it was seen that EVs from *L. major* induced a Th2 response in vivo, causing a more exacerbated infection [16]. For this reason, experiments were carried out to evaluate the expansion of the adaptive response promoted by APC cells challenged by *P. brasiliensis* EVs.

**Figure 6.**
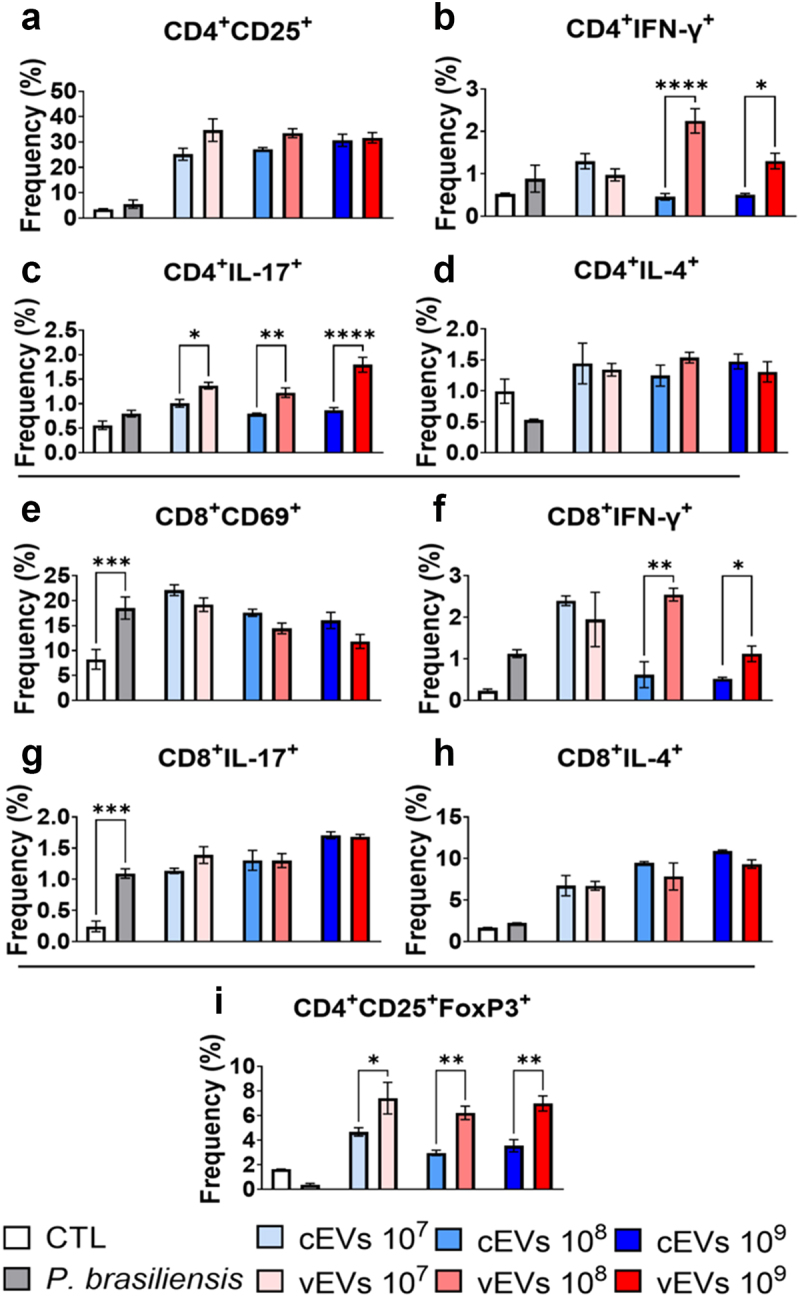
Dendritic cells primed with vEVs induce a higher frequency of Th1/Th17 cells compared to DCs primed with cEVs. Antigen-presenting cells obtained from the bone marrow of C57BL/6 mice, which were differentiated with GM-CSF (20 ng/mL), were challenged with different concentrations of *P. brasiliensis* control and virulent EVs (10^7^, 10^8^, and 10^9^ EVs/mL) for 48 hours. The cells were then placed in co-culture with lymphocytes obtained from the spleen (1 DC/Macrophage : 10 lymphocytes) of naive mice for 5 days. The cells were then recovered and labelled with fluorochrome-conjugated antibodies. Flow cytometry using FACSLyric and FlowJo software was used to analyse the frequency of activated CD4^+^ lymphocytes (a), Th1 (b), Th2 (c), Th17 (d), activated CD8^+^ (e), Tc1 (f), Tc2 (g), Tc17 (h), and Treg (i) profiles. The bars represent means ± standard error of triplicates per group (*p < 0.05; **p < 0.01; ***p < 0.001; ****p < 0.0001). As a negative control (CTL), cells received only RPMI medium, while *P. brasiliensis* (2000 cells; 1 *P. brasiliensis* : 50 innate cells) was used as a positive control.

The results obtained regarding CD4^+^ lymphocytes indicate a cellular adaptive response with an activation profile that showed no significant differences between the groups treated with control and virulent EVs (Figure 6(a)). This observation is supported by the absence of differences in the expression of the CD25 lymphocyte activation marker. However, an increase in Th1 (Figure 6(b)) cells was observed in groups where the innate cells were previously challenged by vEVs when compared to cells exposed to cEVs. Furthermore, a Th17 response (Figure 6(c)) was enhanced solely in the group treated with a concentration of 10^8^ EVs/mL. Additionally, there is no difference in the Th2 cell frequency promoted by cEVs and vEVs (Figure 6(d)). Similarly, the results obtained with CD8^+^ lymphocytes indicate that control and virulent EVs trigger equivalent cytotoxic response (Figure 6(e)), since no difference in the expression of CD69 was founded. However, an increase in Tc1 (Figure 6(f)) frequency was observed in groups which innate cells were previously challenged with vEVs compared to the cells challenged with cEVs. No differences were observed regarding the frequencies of Tc17 (Figure 6(g)) and Tc2 (Figure 6(h)) cells. In addition to the aforementioned adaptive profile, we observed an increase in the frequency of Treg cells in the samples whose innate cells were previously challenged with vEVs (Figure 6(i)), showing that vEVs are responsible for generating a greater regulatory adaptive profile than that promoted by cEVs.

## Discussion

The survival and proliferation of a pathogen are directly linked to the molecules released by them, which have the ability to influence the host’s immune system and facilitate communication between pathogens [7]. EVs serve as carriers for molecules such as proteins, lipids, and RNAs, allowing for the transfer of these substances between the external and internal cellular environments. This function is critical for intercellular communication [8].

Herein, we analysed the proteins found in EVs isolated from a virulent *P. brasiliensis* strain and compared them with the proteome of EVs isolated from a less virulent strain of *P. brasiliensis*. Additionally, we evaluated the EVs’ ability to modulate an immune response in dendritic cells and macrophages. Furthermore, we conducted a pioneering study demonstrating that fungal EVs have the capacity to trigger a cellular adaptive immune response.

When analysing the proteome of vEVs, we observed a higher abundance of certain well-known virulence factors of *P. brasiliensis*, that have been previously found in higher abundance within the virulent yeasts when compared to the control yeasts [19]. For example, glyceraldehyde-3-phosphate dehydrogenase (GAPDH; C1G5F6) and protein 14-3-3 (C1GB04), that are associated with the pathogen adhesion, which has the ability to alter host cell structure and induce macrophage apoptosis [40,41]. In addition, there were important proteins previously recognized as virulence factors in PCM, these proteins were not found in higher abundance in the virulent yeast proteome, but we have found abundant in the vEVs when compared to cEVs. Examples include thioredoxin (C1GE18), associated with the response to oxidative stress, and GP43, which assist in pathogen adhesion [19,36,42]. The fact that those proteins were not in higher abundance in the total yeast proteome, but were in the vEVs in comparison to the cEVs can clarify some aspects of regulation of those virulence factors, since they are modulated by the EVs. Additionally, GP43, the most studied virulence factor in PCM, is known to alter host cell structure, induce macrophage apoptosis, inhibit phagocytosis, and intracellular killing of the fungus [41,43,44]. This glycoprotein has previously been found in extracellular vesicles of *P. brasiliensis* [7], but its higher abundance in vEVs had not been described before. The higher abundance of these proteins in vEVs may explain the increased virulence of these EVs, as evidenced by the reduced activation of DCs and macrophages compared to cells challenged with EVs isolated from the control fungus.

In addition, a higher abundance of proteins that have previously been described as virulence factors for other pathogens, but not for *P. brasiliensis*, was found in the vEVs when compared to the cEVs. The protein with the second highest abundance in vEVs compared to cEVs is aspartyl aminopeptidase, which has been described as a virulence factor for *C. neoformans* [37]. This protein is essential for the growth of *C. neoformans* at 37°C and is related to the nutrient recycling during a nutritional deficiency [37]. The *P. brasiliensis* yeasts within the granuloma undergo nutrient deficiency [19]. Therefore, the greater abundance of aspartyl aminopeptidase in vEVs compared to cEVs raises the speculation that the fungus utilizes its EVs to its advantage. In a previous study conducted by our group, it was reported that *P. brasiliensis* expresses the extracellular siderophore fusarinine C when inside the host as a means of overcoming iron deficiency [19]. Therefore, the presence of the SidJ analogue found exclusively in the vEVs indicates that the virulent fungus improves its capacity to overcome iron deficiency. From this, we can infer that part of the fungal adaptation to high temperature, nutrient deficiency, and iron availability is regulated by extracellular vesicles.

Several proteins related to energy metabolism, lipid metabolism, amino acids, and other metabolic pathways were found with increased concentrations in vEVs when compared to cEVs. Since EVs are associated with cell-to-cell communication within the species, given that they are internalized and their contents utilized [45], we hypothesize that EVs from the virulent fungus have as one of their functions inducing an increase in the metabolic activity and virulence factors of the fungus population as a whole. Previous studies have demonstrated the involvement of *P. brasiliensis* extracellular vesicles as a vehicle for altering fungal cell wall structure [9,10]. In our proteomic assay, proteins related to fungal cell wall remodelling, such as 1,3-beta-glucan synthase, were observed in greater abundance in vEVs than cEVs, highlighting the increased virulence of the cultured granulomatous fungus. Furthermore, extracellular vesicles from *C. neoformans* and *Histoplasma capsulatum* have previously been referred to as “virulence bags,” because they can modulate an immune response in favour of the fungus [46]. Therefore, elucidating the immunomodulatory profile stimulated by extracellular vesicles from *P. brasiliensis* is essential to understand the involvement of EVs in PCM. It also allows us to investigate whether fungal EVs can modulate adaptive responses, which has never been described before.

In fact, experiments were conducted to assess the ability of these EVs to promote different patterns of activation in DCs and macrophages. Although no difference was found in the expression of the co-stimulatory molecule CD40 and the antigen-presenting molecule MHC II in cells challenged with cEVs and vEVs, an impaired expression of the co-stimulatory molecules CD80 and CD86 were observed in DCs and macrophages challenged with vEVs, indicating a compromised maturation of these cells. The reduced expression of co-stimulatory molecules has previously been described in assays with DCs cultured with GP43, where negative regulation of the cells was observed, preventing proper dendritic cell maturation [47,48]. It is worth noting that GP43 was found with increased abundance in the vEVs. Another indication of reduced maturation of DCs challenged with virulent EVs is the impaired expression of TNF-α found in ELISA assays, a cytokine that is crucial for the maturation of these cells [47]. Additionally, the low expression of CD80 and CD86 by macrophages also suggests a reduced M1 macrophage polarization when challenged with vEVs [49]. Furthermore, the increased expression of IL-10 by these cells indicates an anti-inflammatory profile when challenged with vEVs. Along with the lower secretion of TNF-α and MCP1, there is stronger evidence of a less pronounced M1 profile in macrophages challenged with vEVs compared to those challenged with cEVs [50]. It is important to emphasize that although there is indication that the M1 profile is reduced, it should not be interpreted as absent, as it has been described that *P. brasiliensis* EVs can induce this profile in macrophages [11].

A compromised maturation of both DCs and macrophages have been associated with a deleterious and protective effect, since the lower expression of molecules related to antigen presentation can indicate a fungal evasion mechanism [43,48,51]. Instead, in A/J resistant mice, it was shown that the milder activation of DCs and macrophages, resulting in a lower expression of antigen-presenting molecules, is related to a late T-cell response leading to a controlled pathology. This is because an intense activation of the inflammatory response can lead to a poor T-cell mediated immunity [52,53]. In this case, we believe that the milder activation of those cells might be related to an evasion mechanism, since the vEVs were isolated from a more virulent *P. brasiliensis*.

Additionally, a high expression of TLR4 and Dectin-1 associated with a low expression of TLR2 were observed in cells challenged with vEVs. It has been previously described in PCM that pathogen recognition by TLR-4 is actually beneficial for the fungus, as they utilize these receptors to infect the cell and ensure their reproduction [54,55,56,57]. In another study by our group, it was observed that animals and cells deficient in TLR4 promote a more efficient immune response due to a significant reduction in inflammation and an increase in immune response regulation, resulting in lower fungal burdens [55]. Furthermore, the functions of TLR2 and TLR4 are antagonistically involved in the development of Th17 responses, where TLR2 inhibits Th17 immunity and expands the number of Treg cells, while TLR4 induces Th17 cells and inhibits Treg development [56,58,59]. In our assays, an increase in Treg cells was observed in groups whose cells were challenged with vEVs, while an increase in the Th17 response was observed only at higher vEVs concentrations.

A higher production of IL-10 observed in cells challenged with vEVs than cEVs can explain many of the results found so far, as IL-10 suppresses the expression of co-stimulatory molecules like CD80 and CD86 and pro-inflammatory cytokines like TNF-α and GM-CSF, in addition to being responsible for regulating the Th17/Treg adaptive profile [60,61]. Furthermore, IL-10 has already been linked to a more severe PCM, as it is associated with increased susceptibility to the disease [62,63]. In an assay conducted with IL-10 knockout mice, it was observed that there is an improvement in the immune response and regression of the disease in the lungs, liver, and spleen in mice lacking the gene for this cytokine [63]. These data reinforce the hypothesis that vEVs modulate a milder innate inflammatory response. Furthermore, ELISA assays demonstrate a worse chemotaxis and differentiation of innate cells promoted by cells exposed to vEVs, as indicated by low levels of MCP-1 and GM-CSF, respectively. The impaired secretion of these cytokines can be explained by the decreased presence of activated macrophages and DCs expressing CD80 and CD86, which are negatively regulated by increased IL-10 expression.

Additionally, it is known that Treg cells play an important role in the severity of PCM, as both deleterious and protective effects have been shown depending on the experimental model employed [64]. Similarly, the characteristic immunosuppression observed in PCM patients has been associated with a high number of Treg cells expressing FoxP3 in lesions and blood [65]. Of note, the severity of PCM in mice is also affected by the action of myeloid-derived suppressor cells, which contribute to the suppressive microenvironment by producing immunosuppressive molecules and disrupting the activation of T lymphocytes [66,67]. Moreover, it has been described that Treg cell depletion in PCM induces a Th1/Th17 response, associated with a regressive disease with a lower fungal burden [68]. Therefore, the induction of Treg cells by vEVs suggests that these EVs induce a milder inflammatory response compared to cEVs, thus indicating the greater virulence of virulent EVs.

In addition to a higher frequency of Th17 and Treg cells in the groups challenged with vEVs, that can be explained by the increase of IL-6 and IL-10, respectively, an increase in the Th1/Tc1 profiles were also observed in these same groups [60,61,69]. In humans and murine models of PCM, resistance to the disease, often asymptomatic, is associated with the Th1 response [70,71,72]. Although a Th1/Tc1 response were increased, it is known that the fungus recovered from long-term infection is indeed more virulent [19]. Therefore, we can hypothesize that the high frequency of Th1 cells induced by vEVs was not sufficient for a more efficient immune response, given the simultaneous increase in Treg cells and IL-10 expression. Furthermore, the absence of additional stimulation provided by other factors present in vivo that cannot be provided in in vitro environment may also have contributed to the observed results.

The immunomodulatory capacity of *P. brasiliensis* EVs has been previously described by da Silva et al., 2016. In the assays they conducted, it was observed that EVs have the ability to induce an M1-type response in macrophages and were even capable of polarizing M2 macrophages into M1 [11]. Additionally, it was observed that EVs promote the production of pro-inflammatory cytokines in macrophages, such as IL-1β, IL-1α, IL-6, IL-12, and TNF-α. Interestingly, this more virulent profile of EVs obtained from fungi that have undergone infection was recently described by [12] who showed that macrophage cell lines challenged with vEVs express lower amounts of MCP-1, IL-6, and TNF-α compared to cells challenged with EVs from attenuated fungus. Additionally, Octaviano showed that EVs isolated from *P. brasiliensis* exacerbate mice infection when administered 21 days prior to infection. Furthermore, the authors showed that vEVs can enhance virulence traits expression on attenuated *P. brasiliensis* [12]. In *Sporothrix brasiliensis*-infected mice, the fungal burden increases as the number of fungal EVs injected into the mice increased, showing that fungal EVs contribute to the fungal growth within the host [73]. Together with the data presented in this present study, the immunomodulatory capacity of *P. brasiliensis* EVs becomes evident, as well as the higher virulence profile of EVs isolated from fungi that have undergone infection when compared to control fungus. This emphasizes the urge to study the factors present in these extracellular vesicles to discover new virulence mechanisms of the fungus. Interestingly, the use of *P. brasiliensis* EVs along with adjuvants can be used as an immunogen, as the administration of EVs plus adjuvants before animal infection improved T lymphocyte activation, increased secretion of pro-inflammatory molecules, and reduced fungal burden [13].

From what has been presented so far, it is possible to observe that vEVs have increased abundance of well-known virulence factors of *P. brasiliensis* and proteins that have been described as virulence factors in other pathogens. Thus, those vEVs induce a milder activation and maturation of dendritic cells and macrophages. At the same time, there is an increase in an adaptive Th1/Tc1, Th17, as well as a predominant Treg cell profile. However, due to the lower levels of TNF-α found in the groups challenged with vEVs compared to control EVs, it is possible that these EVs are capable of modulating the formation of coalescent granulomas in new infections. This cytokine is crucial for the formation of compact granulomas [72], highlighting the higher degree of virulence of these EVs, since loose granulomas allow for easier dissemination of the fungus to other parts of the lungs and other organs. Additionally, these EVs stimulate greater immunosuppression, as evidenced by the increased presence of Treg cells, and a more severe pathology, as evidenced by increased levels of IL-10. However, at the same time, there is an increase in the Th1/Tc1 and Th17 responses, which are related to a more efficient immune response in PCM. In any case, the fungus that has undergo through long-term infections exhibits greater virulence and lethality, and can even induce looser granulomas and higher dissemination than fungus that have not undergone infection, as demonstrated by previous reports [19]. Furthermore, it is known that EVs enhance the infection of *P. brasiliensis* [12]. Therefore, the hypothesis that *P. brasiliensis* EVs act as virulence bags that manipulate the immune system in their favour is strengthened.

## Supplementary Material

Supplemental Material

## Data Availability

Proteome data are available via ProteomeXchange with identifier PXD046549. Any other data are available upon request.
