# Supplementary material for: Extracellular vesicles from virulent P. brasiliensis induce TLR4 and dectin-1 expression in innate cells and promote enhanced Th1/Th17 response
Source: Virulence. 2024 Mar 21;15(1):2329573. doi: 10.1080/21505594.2024.2329573 (PMC10962619; doi:10.1080/21505594.2024.2329573)
Supplement: Supplemental Material [file KVIR_A_2329573_SM2101.zip › Supplementary Table 1.docx]

**Supplementary Table 1.**

| **Acession number** | **Protein** | **Expression status** | **Log (Fold change)** | **Adjusted p-Value** |
| --- | --- | --- | --- | --- |
| **Virulence factor** | |  |  |  |
| C1GBY9 | Allergen Asp f 7 homolog | UP (8S) | 6,012732 | 0,041254 |
| C1GDK8 | Thioredoxin domain-containing protein | UP (8S) | 5,613289 | 0,02906 |
| C1G7L4 | alpha-1,3-glucan synthase | UP (8S) | 5,083855 | 0,020855 |
| C1G5W4 | Yeast cell wall synthesis Kre9/Knh1-like N-terminal domain-containing protein | UP (12S) | 5,06516 | 0,025008 |
| C1GE18 | Thioredoxin | UP(D) | 4,131451 | 0,000105 |
| C1GK34 | Yeast cell wall synthesis Kre9/Knh1-like N-terminal domain-containing protein | UP(D) | 3,581609 | 0,000173 |
| C1G6F9 | Thioredoxin-like_fold domain-containing protein | UP(D) | 3,416249 | 0,000246 |
| C1GKN3 | Endo-1,3(4)-beta-glucanase | UP (8S) | 3,029346 | 0,04212 |
| C1GB04 | 14-3-3 protein epsilon | UP(D) | 2,719102 | 4,88E-05 |
| C1GK29 | Glucan 1,3-beta-glucosidase | UP(D) | 2,596273 | 0,000332 |
| A0A0A0HS55 | 1,3-beta-glucan synthase | UP(D) | 2,298877 | 0,000759 |
| C1G5F6 | Glyceraldehyde-3-phosphate dehydrogenase | UP(D) | 2,169445 | 0,000128 |
| C1G0D4 | Catalase | UP (8S) | 1,950176 | 0,033535 |
| **Gene/protein regulation** | | |  |  |
| C1G5P6 | PWWP domain-containing protein | UP (8S) | 7,212353 | 0,038532 |
| C1GG06 | Camp independent regulatory protein | UP (8S) | 6,261013 | 0,023829 |
| C1GKX0 | Eukaryotic translation initiation factor 4C | UP (12S) | 6,097781 | 0,044692 |
| C1G4I2 | Protein HIR1 | UP (8S) | 6,00221 | 0,019701 |
| C1G651 | Vacuolar protein sorting-associated protein 17 | UP (12S) | 5,963711 | 0,029066 |
| A0A0A0HUT3 | Replication factor C subunit 4 | UP (12S) | 5,885317 | 0,025008 |
| C1GBE7 | H/ACA ribonucleoprotein complex subunit GAR1 | UP (8S) | 5,714351 | 0,038532 |
| C1GGX0 | Elongator complex protein 2 | UP(D) | 5,5947 | 3,89E-05 |
| C1G8X4 | Ppx/GppA phosphatase domain-containing protein | UP(D) | 5,561267 | 3,44E-05 |
| C1GD24 | Dipeptidyl peptidase 3 | UP (12S) | 5,560135 | 0,025036 |
| C1G4M6 | Pre-mRNA-processing factor 40 | UP (8S) | 5,480455 | 0,019701 |
| C1G9U5 | tRNA (guanine-N(7)-)-methyltransferase non-catalytic subunit TRM82 | UP (12S) | 5,466421 | 0,037052 |
| C1G8V3 | tyrosyl-tRNA synthetase [EC:6.1.1.1] | UP(D) | 5,396857 | 5,91E-05 |
| C1GLC7 | transcriptional activator HAP5 | UP (8S) | 5,360828 | 0,032085 |
| C1G798 | Pescadillo homolog | UP (8S) | 5,312812 | 0,026402 |
| C1GDQ7 | Scavenger mRNA decapping enzyme | UP(D) | 5,295471 | 0,000405 |
| C1G1Q6 | Armadillo repeat-containing protein 8 | UP (8S) | 4,98412 | 0,046006 |
| C1GEM5 | 5'-3' exoribonuclease 1 | UP (8S) | 4,839948 | 0,020232 |
| C1G0P0 | Hsp7-like protein | UP(D) | 4,759801 | 5,66E-05 |
| C1GM86 | N-acetyltransferase domain-containing protein | UP (8S) | 4,743161 | 0,025741 |
| C1G787 | Glycylpeptide N-tetradecanoyltransferase | UP (8S) | 4,738889 | 0,033535 |
| C1GD78 | Ubiquitin thiolesterase | UP (8S) | 4,72224 | 0,049147 |
| C1G746 | N-acetyltransferase domain-containing protein | UP (8S) | 4,570015 | 0,038532 |
| C1GL41 | T-complex protein 1 subunit beta | UP (8S) | 4,486968 | 0,019701 |
| A0A0A0HR72 | Sorting nexin-41 | UP(D) | 4,472729 | 9,33E-05 |
| C1GDK4 | RRM domain-containing protein | UP(D) | 4,418129 | 9,12E-05 |
| C1FZK4 | 28 kDa ribonucleoprotein | UP (8S) | 4,400977 | 0,019701 |
| C1GAD5 | Xpo1 domain-containing protein | UP (8S) | 4,312873 | 0,040134 |
| C1G2H4 | methionine--tRNA ligase | UP (8S) | 4,304465 | 0,020855 |
| A0A0A0HY23 | Cleavage and polyadenylation specificity factor subunit 5 | UP(D) | 4,2703 | 8,87E-05 |
| C1GG78 | SBDS family rRNA metabolism protein | UP (8S) | 4,242292 | 0,02906 |
| C1GFD8 | IEC3 subunit of the Ino80 complex, chromatin re-modelling-domain-containing protein | UP (8S) | 4,215421 | 0,028828 |
| C1G3Y0 | RuvB-like helicase | UP (8S) | 4,185007 | 0,019701 |
| C1GG12 | glutamine--tRNA ligase | UP(D) | 4,162841 | 3,44E-05 |
| C1G5J0 | 40S ribosomal protein S15 | UP (8S) | 4,13502 | 0,020855 |
| C1G9R5 | [RNA-polymerase]-subunit kinase | UP (8S) | 4,132251 | 0,030549 |
| C1GA10 | ATP-dependent RNA helicase DDX3X [EC:3.6.4.13] | UP (8S) | 4,084858 | 0,022833 |
| C1FZL0 | T-complex protein 1 subunit delta | UP (8S) | 4,048354 | 0,019701 |
| C1GIL9 | arginyl-tRNA synthetase [EC:6.1.1.19] | UP(D) | 4,047925 | 6,5E-05 |
| C1GKB4 | Exportin-T | UP (12S) | 4,046354 | 0,028417 |
| C1GK92 | Splicing factor U2AF subunit | UP (8S) | 4,012214 | 0,019701 |
| C1GKU8 | 3' exoribonuclease | UP (12S) | 4,006895 | 0,025008 |
| C1G5P0 | Pre-mRNA-splicing factor brr2 | UP(D) | 3,976934 | 6,85E-05 |
| C1FYE8 | HABP4_PAI-RBP1 domain-containing protein | UP(D) | 3,97431 | 0,000753 |
| A0A0A0HVY3 | Cullin-1 | UP(D) | 3,92781 | 5,67E-05 |
| C1GL49 | replication factor A1 | UP(D) | 3,927564 | 8,23E-05 |
| C1G017 | Zuotin | UP (8S) | 3,825741 | 0,023829 |
| C1G695 | ATP-dependent DNA helicase II subunit 1 | UP(D) | 3,817383 | 4,39E-05 |
| C1GDP1 | RNA binding protein Jsn1 | UP (12S) | 3,785346 | 0,033583 |
| C1G371 | 60S ribosomal protein L7 | UP (8S) | 3,750258 | 0,021657 |
| C1G3D9 | K Homology domain-containing protein | UP(D) | 3,726014 | 5,33E-05 |
| C1GLS6 | Ubiquitin conjugation factor E4 B | UP(D) | 3,723561 | 8,82E-05 |
| C1G7R0 | Importin N-terminal domain-containing protein | UP (12S) | 3,706996 | 0,025036 |
| C1G8Q6 | Tryptophan--tRNA ligase | UP (8S) | 3,701979 | 0,041998 |
| C1G306 | Cell division control protein 73 C-terminal domain-containing protein | UP (8S) | 3,652095 | 0,019701 |
| C1GF61 | seryl-tRNA synthetase [EC:6.1.1.11] | UP(D) | 3,637835 | 9,12E-05 |
| C1GIS6 | U6 snRNA-associated Sm-like protein LSm4 | UP(D) | 3,631133 | 4,39E-05 |
| C1G435 | GPI mannosyltransferase 2 | UP(D) | 3,616139 | 3,44E-05 |
| C1FZ06 | NA | UP(D) | 3,549867 | 3,44E-05 |
| C1G7J2 | Pre-mRNA-processing factor 39 | UP(D) | 3,548202 | 0,000276 |
| C1GJL2 | cysteinyl-tRNA synthetase [EC:6.1.1.16] | UP(D) | 3,526538 | 4,92E-05 |
| C1GAW5 | Pre-mRNA-processing-splicing factor 8 | UP(D) | 3,500051 | 9,12E-05 |
| C1GK84 | SAP domain-containing protein | UP(D) | 3,498052 | 0,000331 |
| C1GGW1 | MIF4G domain-containing protein | UP(D) | 3,464096 | 4,11E-05 |
| C1G8U3 | DNA-directed RNA polymerase subunit beta | UP (8S) | 3,458519 | 0,035344 |
| C1G4M5 | Translation initiation factor RLI1 | UP (8S) | 3,458238 | 0,024461 |
| C1G9X1 | Eukaryotic translation initiation factor 3 subunit J | UP (8S) | 3,454026 | 0,019701 |
| C1FYE5 | glutamyl-tRNA synthetase [EC:6.1.1.17] | UP(D) | 3,449056 | 0,000124 |
| C1G194 | Vacuolar protease A | UP(D) | 3,441545 | 4,33E-05 |
| C1GLA9 | translation initiation factor 5B | UP (8S) | 3,437828 | 0,029573 |
| C1G554 | ubiquitin-activating enzyme E1 [EC:6.2.1.45] | UP(D) | 3,433079 | 6,85E-05 |
| C1G1H8 | histone chaperone ASF1 | UP(D) | 3,419422 | 4,88E-05 |
| C1G3P2 | histidine--tRNA ligase | UP(D) | 3,417428 | 4,51E-05 |
| C1GBG1 | aspartyl-tRNA synthetase [EC:6.1.1.12] | UP(D) | 3,403972 | 9,12E-05 |
| C1GI12 | RRM domain-containing protein | UP (8S) | 3,400077 | 0,022455 |
| C1GCV8 | T-complex protein 1 subunit zeta | UP(D) | 3,383136 | 8,66E-05 |
| C1G3P9 | Calnexin | UP (12S) | 3,374269 | 0,048674 |
| C1GH47 | tRNA pseudouridine13 synthase | UP(D) | 3,362984 | 0,000235 |
| C1GAK2 | Endoribonuclease L-PSP | UP (12S) | 3,359172 | 0,025036 |
| C1G9X0 | DNA damage checkpoint protein rad24 | UP(D) | 3,352729 | 4,11E-05 |
| A0A0A0HTF2 | tRNA ligase | UP (12S) | 3,350342 | 0,025008 |
| C1G4I3 | obg-like ATPase 1 | UP(D) | 3,346715 | 9,12E-05 |
| C1G514 | Hsp90 chaperone protein kinase-targeting subunit | UP(D) | 3,341308 | 6,78E-05 |
| C1GHV0 | DMAP1-binding domain-containing protein | UP (8S) | 3,330404 | 0,023878 |
| C1GAG5 | translation initiation factor 3 subunit I | UP (8S) | 3,33018 | 0,022455 |
| C1GF39 | H/ACA ribonucleoprotein complex subunit CBF5 | UP(D) | 3,328386 | 0,001219 |
| C1G570 | prolyl-tRNA synthetase [EC:6.1.1.15] | UP(D) | 3,300397 | 0,000152 |
| C1G8B2 | t-SNARE coiled-coil homology domain-containing protein | UP (8S) | 3,294465 | 0,029047 |
| C1GDQ2 | Asparagine--tRNA ligase | UP(D) | 3,276908 | 0,000111 |
| C1G9Z7 | translation initiation factor 2 subunit 3 | UP (8S) | 3,271376 | 0,022937 |
| C1GJI0 | Probable endonuclease LCL3 | UP (8S) | 3,261387 | 0,020855 |
| C1G632 | ubiquitin-conjugating enzyme (huntingtin interacting protein 2) [EC:2.3.2.23] | UP (12S) | 3,248563 | 0,045762 |
| C1G561 | aminoacyl tRNA synthase complex-interacting multifunctional protein 1 | UP(D) | 3,218509 | 9,12E-05 |
| C1GF01 | E3 ubiquitin ligase complex SCF subunit | UP(D) | 3,217103 | 0,000196 |
| C1G4K1 | HMG box domain-containing protein | UP(D) | 3,216911 | 0,000106 |
| C1GIP8 | Histone H4 | UP (12S) | 3,203145 | 0,0276 |
| C1G5M9 | valyl-tRNA synthetase [EC:6.1.1.9] | UP(D) | 3,188581 | 4,51E-05 |
| C1G6R0 | protein SSD1 | UP (12S) | 3,187648 | 0,0458 |
| C1GN64 | HECT-type E3 ubiquitin transferase | UP (8S) | 3,182105 | 0,037294 |
| C1GL26 | translation initiation factor 3 subunit B | UP (8S) | 3,178496 | 0,023829 |
| C1G6I0 | Hsp88-like protein | UP(D) | 3,177862 | 3,44E-05 |
| C1GBD3 | RRM domain-containing protein | UP(D) | 3,176773 | 4,39E-05 |
| A0A0A0HYV6 | 60S ribosomal protein L5 | UP (12S) | 3,173867 | 0,025008 |
| C1GEQ8 | Alpha-1,2 mannosyltransferase KTR1 | UP(D) | 3,171146 | 0,000156 |
| C1GCS4 | Pre-mRNA-splicing factor rse1 | UP(D) | 3,165448 | 4,39E-05 |
| C1GIS0 | Histone acetyltransferase type B catalytic subunit | UP(D) | 3,157724 | 9,61E-05 |
| C1GKL7 | 60S ribosomal protein L12 | UP(D) | 3,152401 | 4,11E-05 |
| C1G9A5 | protein disulfide-isomerase A1 [EC:5.3.4.1] | UP (12S) | 3,149511 | 0,029047 |
| C1GM02 | Mitochondrial DNA replication protein YHM2 | UP (12S) | 3,143745 | 0,025008 |
| C1GF71 | Histone H2B | UP (12S) | 3,128918 | 0,0458 |
| C1G7J4 | Aminopeptidase | UP(D) | 3,124206 | 8,23E-05 |
| C1FZG2 | Alanine--tRNA ligase | UP(D) | 3,121169 | 9,12E-05 |
| C1GMV5 | DnaJ domain protein | UP (8S) | 3,111922 | 0,031909 |
| A0A0A0HZD4 | RNA (Cytosine-5-)-methyltransferase NCL1 | UP (8S) | 3,107829 | 0,045621 |
| C1G8H6 | Endoplasmic reticulum chaperone BiP | UP(D) | 3,103103 | 0,000208 |
| A0A0A0HT52 | GTP cyclohydrolase | UP(D) | 3,098535 | 5,48E-05 |
| C1GF18 | Tetratricopeptide repeat protein 1 | UP (8S) | 3,096709 | 0,026127 |
| C1GG28 | Elongation factor 1-gamma | UP(D) | 3,085752 | 8,23E-05 |
| C1G2H7 | MYG1 protein | UP(D) | 3,082804 | 9,12E-05 |
| C1GL98 | polyadenylate-binding protein | UP(D) | 3,081258 | 4,39E-05 |
| C1G4C0 | N-alpha-acetyltransferase 15/16, NatA auxiliary subunit | UP (8S) | 3,073082 | 0,038221 |
| C1G186 | Eukaryotic translation initiation factor 3 subunit E | UP (8S) | 3,070495 | 0,022455 |
| A0A0A0HU09 | Poly(RC)-binding protein | UP(D) | 3,067462 | 4,39E-05 |
| C1G4P8 | 60S ribosomal protein L35 | UP (8S) | 3,035991 | 0,022455 |
| C1G9T0 | translation initiation factor 3 subunit A | UP (8S) | 3,033381 | 0,021908 |
| A0A0A0HV97 | Transcription initiation factor TFIID/TFIIF subunit | UP (12S) | 3,030708 | 0,025008 |
| C1G2D4 | Protein phosphatase | UP(D) | 3,028293 | 0,000135 |
| C1GFG5 | Histone H1 | UP (8S) | 3,024314 | 0,028431 |
| C1GJZ6 | Phenylalanine--tRNA ligase | UP(D) | 3,021804 | 4,33E-05 |
| C1GB62 | leucyl-tRNA synthetase [EC:6.1.1.4] | UP(D) | 3,017328 | 6,85E-05 |
| C1GIZ3 | Elongation factor 3 | UP(D) | 3,0154 | 0,000208 |
| C1G4Y3 | Small nuclear ribonucleoprotein Sm D3 | UP(D) | 3,010427 | 0,00011 |
| C1G1M5 | ATP-dependent Clp protease ATP-binding subunit ClpB | UP (8S) | 3,007447 | 0,020855 |
| C1GFL5 | PCI domain-containing protein | UP (12S) | 2,984588 | 0,032289 |
| C1GJX2 | Ubiquitinyl hydrolase 1 | UP (8S) | 2,963042 | 0,021931 |
| C1GM32 | RuvB-like helicase 2 | UP (12S) | 2,9494 | 0,033901 |
| A0A0A0HQN9 | 40S ribosomal protein S19 | UP(D) | 2,93814 | 4,88E-05 |
| C1FZP0 | Eukaryotic translation initiation factor 3 subunit D | UP (8S) | 2,932412 | 0,026668 |
| C1GKS2 | Ubiquitin carboxyl-terminal hydrolase | UP(D) | 2,930768 | 9,06E-05 |
| C1GF43 | Eukaryotic peptide chain release factor subunit 1 | UP (12S) | 2,922704 | 0,031427 |
| C1G6T3 | 60S ribosomal protein L6 | UP (8S) | 2,903179 | 0,021908 |
| C1GEW2 | SsDNA binding protein | UP(D) | 2,882354 | 8,87E-05 |
| C1GK99 | 40S ribosomal protein | UP (8S) | 2,877558 | 0,026668 |
| C1GLD3 | FACT complex subunit SPT16 | UP (8S) | 2,87026 | 0,023829 |
| C1G6E7 | 116 kDa U5 small nuclear ribonucleoprotein component | UP (8S) | 2,869792 | 0,03113 |
| C1GIJ9 | Transcriptional adapter 2-alpha | UP(D) | 2,863618 | 0,000322 |
| C1GJM4 | Aspartyl aminopeptidase | UP (12S) | 2,85516 | 0,025008 |
| A0A0A0HUX3 | Proliferating cell nuclear antigen | UP(D) | 2,849172 | 8,3E-05 |
| C1GF12 | glycyl-tRNA synthetase [EC:6.1.1.14] | UP(D) | 2,838008 | 4,39E-05 |
| C1GBI7 | NEDD8-activating enzyme E1 regulatory subunit | UP(D) | 2,834605 | 0,000259 |
| C1GF47 | 60S ribosomal protein L25 | UP (12S) | 2,821853 | 0,031333 |
| C1G373 | translation initiation factor 3 subunit C | UP (8S) | 2,786963 | 0,021452 |
| C1GE31 | Rho GDP-dissociation inhibitor | UP(D) | 2,774396 | 0,000476 |
| A0A0A0HTY8 | nucleolar protein 58 | UP (8S) | 2,755448 | 0,045621 |
| C1GLI2 | Heat shock 70 kDa protein 2 | UP(D) | 2,71695 | 8,65E-05 |
| C1FYK2 | isoleucine--tRNA ligase | UP(D) | 2,701848 | 0,00011 |
| A0A0A0HSU0 | Insulysin | UP(D) | 2,70003 | 0,000211 |
| A0A0A0HVL0 | Putative transcription factor kapC | UP (8S) | 2,684034 | 0,033986 |
| C1GGQ1 | PPIase cyclophilin-type domain-containing protein | UP(D) | 2,66914 | 0,000631 |
| C1GN83 | CMGC/GSK protein kinase | UP (12S) | 2,656696 | 0,0458 |
| C1FZD4 | Eukaryotic translation initiation factor 3 subunit M | UP (8S) | 2,632386 | 0,028663 |
| C1G2D0 | Eukaryotic translation initiation factor 3 subunit F | UP (8S) | 2,630899 | 0,026402 |
| C1G678 | UV excision repair protein RAD23 | UP(D) | 2,613846 | 0,000456 |
| C1G9A3 | Uracil-DNA glycosylase | UP(D) | 2,611536 | 0,000102 |
| C1G172 | large subunit ribosomal protein L27e | UP(D) | 2,605768 | 9,24E-05 |
| C1G8W4 | E3 ubiquitin-protein ligase | UP(D) | 2,603044 | 0,000207 |
| C1GKL8 | protein transport protein SEC23 | UP (8S) | 2,590148 | 0,022455 |
| C1GH63 | 40S ribosomal protein S25 | UP (8S) | 2,585824 | 0,041482 |
| C1FZM4 | tRNA (Guanine37-N1)-methyltransferase | UP (12S) | 2,583133 | 0,025901 |
| A0A0A0HUI8 | Histone H2A.Z | UP (8S) | 2,57407 | 0,040657 |
| C1GEM3 | Histone acetyltransferase type B subunit 2 | UP (8S) | 2,560113 | 0,028317 |
| C1G5H3 | Protein CFT1 | UP (12S) | 2,557585 | 0,040086 |
| C1GFA3 | 60S ribosomal protein L27a | UP (8S) | 2,556447 | 0,024792 |
| C1G390 | phenylalanyl-tRNA synthetase beta chain [EC:6.1.1.20] | UP(D) | 2,542429 | 9,37E-05 |
| C1GG39 | tRNA N6-adenosine threonylcarbamoyltransferase | UP (12S) | 2,538047 | 0,029047 |
| C1GAU3 | STI1 domain-containing protein | UP (8S) | 2,526339 | 0,021657 |
| A0A0A0HWY2 | Ribosomal protein L6 alpha-beta domain-containing protein | UP (8S) | 2,510757 | 0,036583 |
| C1GLX8 | Heat shock protein 60, mitochondrial | UP(D) | 2,503694 | 0,000225 |
| C1G293 | Adenosinetriphosphatase | UP(D) | 2,49195 | 0,000323 |
| C1G6F6 | heat shock 70kDa protein 1/2/6/8 | UP(D) | 2,485377 | 0,00031 |
| C1G0P8 | Eukaryotic translation initiation factor 3 subunit G | UP (8S) | 2,483635 | 0,044593 |
| C1G3N7 | P/Homo B domain-containing protein | UP (8S) | 2,467017 | 0,040877 |
| C1GD28 | protein transport protein SEC13 | UP (8S) | 2,460744 | 0,037729 |
| C1GLF9 | 26S proteasome regulatory subunit rpn-8 | UP(D) | 2,457599 | 0,00064 |
| C1GH70 | ribonuclease Z | UP (8S) | 2,450583 | 0,030267 |
| C1FYQ2 | T-complex protein 1 subunit gamma | UP (8S) | 2,441847 | 0,023829 |
| C1G0B0 | 26S protease regulatory subunit 8 | UP (8S) | 2,431942 | 0,022455 |
| C1GC66 | 60S ribosomal protein L22 | UP(D) | 2,430412 | 8,23E-05 |
| C1GAQ4 | DNA-directed RNA polymerase II subunit RPB2 [EC:2.7.7.6] | UP (8S) | 2,407605 | 0,028317 |
| C1G945 | large subunit ribosomal protein L30e | UP (8S) | 2,390311 | 0,023537 |
| C1G352 | AGC/AKT protein kinase | UP (12S) | 2,389203 | 0,040495 |
| C1GKC9 | Hsp90-like protein | UP(D) | 2,384169 | 0,000121 |
| C1GN20 | DNA mismatch repair protein MSH3 | UP (12S) | 2,38317 | 0,033162 |
| C1G9U4 | 60S acidic ribosomal protein P0 | UP(D) | 2,368157 | 0,000149 |
| C1GAM1 | RRM domain-containing protein | UP(D) | 2,364084 | 0,000744 |
| C1G820 | 60S ribosomal protein L21-A | UP (8S) | 2,359968 | 0,030726 |
| C1GA62 | alpha-mannosidase [EC:3.2.1.24] | UP(D) | 2,344228 | 0,000107 |
| C1GEA1 | U2 small nuclear ribonucleoprotein B | UP(D) | 2,306375 | 0,000379 |
| C1GBE8 | Threonine--tRNA ligase | UP (8S) | 2,287815 | 0,024792 |
| C1G9Z0 | Proteasome subunit beta | UP (12S) | 2,286281 | 0,035547 |
| C1GIV4 | Proteasome subunit alpha type | UP(D) | 2,28346 | 7,42E-05 |
| C1G9Y1 | Proteasome subunit beta | UP(D) | 2,257783 | 0,000114 |
| C1FZI7 | Vacuolar protein sorting/targeting protein 10 | UP(D) | 2,248742 | 0,000121 |
| C1GG53 | Proteasome alpha-type subunits domain-containing protein | UP(D) | 2,2336 | 0,000173 |
| C1GCK8 | Nascent polypeptide-associated complex subunit alpha | UP(D) | 2,229065 | 9,12E-05 |
| C1GIL5 | Serine/threonine-protein kinase RIO1 | UP (12S) | 2,226983 | 0,03073 |
| C1G3L4 | Guanine nucleotide-binding protein subunit beta-like protein | UP (12S) | 2,220474 | 0,033901 |
| C1G175 | Proteasome subunit alpha type | UP (12S) | 2,19911 | 0,028963 |
| C1G6U1 | Elongation factor 1-beta | UP (8S) | 2,184333 | 0,033289 |
| C1G9P6 | PROTEASOME_ALPHA_1 domain-containing protein | UP(D) | 2,177669 | 0,000114 |
| C1GLJ3 | T-complex protein 1 subunit eta | UP (8S) | 2,169971 | 0,036086 |
| C1GMI6 | lysyl-tRNA synthetase, class II [EC:6.1.1.6] | UP (12S) | 2,167479 | 0,049928 |
| C1G7N7 | Proteasome component PUP2 | UP (12S) | 2,145423 | 0,031427 |
| C1G1F2 | Elongation factor 1-alpha | UP (8S) | 2,142863 | 0,027277 |
| C1G6M3 | Ribosomal protein | UP(D) | 2,140685 | 0,000173 |
| C1G391 | small subunit ribosomal protein S3e | UP (8S) | 2,12023 | 0,037729 |
| C1G5J1 | 60S acidic ribosomal protein P2 | UP(D) | 2,118248 | 0,000123 |
| C1G9K2 | Serine/threonine protein kinase | UP (8S) | 2,108311 | 0,040134 |
| C1FYJ6 | Histone H4 | UP (12S) | 2,094548 | 0,036284 |
| C1GLI9 | Elongation factor 2 | UP(D) | 2,08962 | 0,000274 |
| C1G0F2 | Eukaryotic translation initiation factor 3 subunit L | UP (8S) | 2,087353 | 0,033325 |
| C1G3B1 | 40S ribosomal protein S12 | UP (12S) | 2,026207 | 0,032773 |
| C1G571 | T-complex protein 1 subunit alpha | UP (8S) | 1,994393 | 0,038532 |
| C1GHB0 | Ubiquitin carboxyl-terminal hydrolase | UP (12S) | 1,975931 | 0,036284 |
| C1G733 | Mitotic control protein dis3 | UP(D) | 1,975227 | 0,000309 |
| C1GF57 | nucleosome assembly protein 1-like 1 | UP (12S) | 1,945934 | 0,036996 |
| C1G8U4 | Proteasome subunit beta | UP(D) | 1,924127 | 0,000208 |
| C1G9N1 | 20S proteasome subunit beta 6 | UP(D) | 1,858894 | 0,000295 |
| C1G6T2 | Pru domain-containing protein | UP (8S) | 1,769032 | 0,045584 |
| C1GMF6 | Proteasome subunit alpha type | UP (12S) | 1,764216 | 0,049928 |
| C1GEN5 | large subunit ribosomal protein L4e | UP (8S) | 1,746114 | 0,038992 |
| C1G6D0 | Proteasome subunit alpha type | UP (12S) | 1,744521 | 0,041366 |
| A0A0A0HVT2 | AAA+ ATPase domain-containing protein | UP (8S) | 1,717681 | 0,040877 |
| **Energy metabolism** | | |  |  |
| C1GI92 | Cytochrome c | UP (8S) | 8,920571 | 0,020855 |
| C1GL50 | NADPH2:quinone reductase | UP(D) | 6,671233 | 5,33E-05 |
| C1GAD3 | Glycosyl hydrolase | UP(D) | 5,72561 | 8,69E-05 |
| C1GAH8 | Beta-mannosidase | UP(D) | 5,649712 | 0,000105 |
| A0A0A0HUM5 | Aconitate hydratase, mitochondrial | UP (12S) | 5,506735 | 0,046613 |
| C1G5V6 | ATP synthase subunit alpha | UP (8S) | 5,324719 | 0,019701 |
| C1GBI8 | Ribose-5-phosphate isomerase | UP(D) | 5,144232 | 3,44E-05 |
| C1G496 | Oxoglutarate dehydrogenase (succinyl-transferring) | UP (8S) | 4,566625 | 0,040068 |
| C1GL74 | Beta-hexosaminidase | UP(D) | 4,443754 | 0,000207 |
| C1G2P3 | Enoyl-CoA hydratase | UP(D) | 4,382212 | 3,44E-05 |
| C1G977 | Hexokinase | UP (12S) | 4,342947 | 0,032162 |
| A0A0A0HX82 | phosphoglucomutase [EC:5.4.2.2] | UP(D) | 4,23921 | 4,39E-05 |
| C1GKU3 | Glucokinase | UP (12S) | 4,13475 | 0,025008 |
| C1G1C8 | Fructose-bisphosphate aldolase 1 | UP (12S) | 4,082478 | 0,02646 |
| C1GCI8 | 2-methylcitrate synthase, mitochondrial | UP(D) | 3,832717 | 4,39E-05 |
| C1G4D1 | Dihydrolipoamide acetyltransferase component of pyruvate dehydrogenase complex | UP(D) | 3,765113 | 6,85E-05 |
| C1GAB4 | Glucose-6-phosphate 1-dehydrogenase | UP(D) | 3,666746 | 4,39E-05 |
| C1GLI3 | fumarate hydratase, class II [EC:4.2.1.2] | UP (12S) | 3,624739 | 0,035325 |
| C1G547 | Pyruvate dehydrogenase E1 component subunit alpha | UP (8S) | 3,622414 | 0,022455 |
| C1G0R1 | Glucose-6-phosphate isomerase | UP (12S) | 3,508344 | 0,031334 |
| C1G6E6 | acetyl-CoA C-acetyltransferase [EC:2.3.1.9] | UP (8S) | 3,469434 | 0,020855 |
| C1G440 | fructose-1,6-bisphosphatase I [EC:3.1.3.11] | UP (8S) | 3,41363 | 0,024814 |
| C1GIX7 | Acetyltransferase component of pyruvate dehydrogenase complex | UP(D) | 3,376048 | 0,0002 |
| C1GCX3 | 2,3-bisphosphoglycerate-independent phosphoglycerate mutase [EC:5.4.2.12] | UP (12S) | 3,232137 | 0,031427 |
| C1FZY1 | L-lactate dehydrogenase (Cytochrome) | UP (8S) | 3,170088 | 0,023878 |
| C1GL12 | Glycogen debranching enzyme | UP(D) | 2,979608 | 0,000124 |
| C1GE37 | NADPH2:quinone reductase | UP(D) | 2,959892 | 0,000476 |
| C1GI20 | triosephosphate isomerase (TIM) [EC:5.3.1.1] | UP(D) | 2,934777 | 0,000225 |
| C1G3G7 | Isocitrate lyase | UP (12S) | 2,890295 | 0,033162 |
| C1G8R5 | 6-phosphogluconate dehydrogenase [EC:1.1.1.44 1.1.1.343] | UP(D) | 2,813089 | 6,85E-05 |
| C1GKG3 | NAD(P)H:quinone oxidoreductase, type IV | UP (8S) | 2,723812 | 0,045621 |
| C1G4N0 | Phosphoglycerate kinase | UP(D) | 2,717641 | 0,000213 |
| C1G2W2 | Pyruvate kinase | UP (8S) | 2,58962 | 0,021657 |
| C1GJI4 | Transaldolase | UP(D) | 2,540403 | 0,000111 |
| C1GKQ0 | Pyruvate decarboxylase | UP(D) | 2,501055 | 0,000124 |
| C1GAG3 | Isocitrate dehydrogenase [NADP] | UP (8S) | 2,432521 | 0,028362 |
| C1G9P1 | Isocitrate dehydrogenase (NAD(+)) | UP (8S) | 2,370944 | 0,042671 |
| C1G1H4 | pyruvate decarboxylase [EC:4.1.1.1] | UP (8S) | 2,305338 | 0,023769 |
| C1GMZ1 | Peroxisomal hydratase-dehydrogenase-epimerase | UP (8S) | 2,28588 | 0,027458 |
| C1G9X3 | Enolase | UP(D) | 2,25336 | 0,000323 |
| C1G002 | 6-phosphofructokinase 1 [EC:2.7.1.11] | UP (8S) | 2,237191 | 0,023829 |
| C1G5Y7 | Cytochrome b5 heme-binding domain-containing protein | UP (12S) | 1,831007 | 0,042742 |
| C1G1T5 | Glutamate synthase (NADH) | UP (8S) | 1,757421 | 0,046227 |
| **Amino acid metabolism** | | |  |  |
| C1GN18 | ATP phosphoribosyltransferase | UP (8S) | 7,51305 | 0,024044 |
| C1GG15 | 1,2-dihydroxy-3-keto-5-methylthiopentene dioxygenase | UP (12S) | 7,10574 | 0,026267 |
| C1GJ05 | dihydroxy-acid dehydratase [EC:4.2.1.9] | UP (12S) | 6,690133 | 0,025655 |
| C1G452 | saccharopine dehydrogenase (NADP+, L-glutamate forming) [EC:1.5.1.10] | UP (8S) | 5,157285 | 0,021908 |
| C1GLM1 | asparagine synthase (glutamine-hydrolysing) [EC:6.3.5.4] | UP (12S) | 4,901568 | 0,0458 |
| C1G4J8 | Kynurenine formamidase | UP (12S) | 4,556819 | 0,04046 |
| C1G3Q0 | Methylthioribulose-1-phosphate dehydratase | UP(D) | 4,193475 | 5,07E-05 |
| C1GF86 | Cys-Gly metallodipeptidase DUG1 [EC:3.4.13.-] | UP(D) | 4,12676 | 0,000208 |
| C1G6H2 | threonine synthase [EC:4.2.3.1] | UP(D) | 3,898842 | 8,58E-05 |
| C1G6P2 | 2,4-dihydroxyhept-2-ene-1,7-dioic acid aldolase | UP(D) | 3,82139 | 4,39E-05 |
| C1G838 | GS catalytic domain-containing protein | UP(D) | 3,687192 | 7,85E-05 |
| C1GK23 | Dihydroxy-acid dehydratase | UP (12S) | 3,633367 | 0,025008 |
| C1GMH9 | Fumarylacetoacetase | UP (12S) | 3,623618 | 0,037387 |
| C1G0F9 | kynureninase [EC:3.7.1.3] | UP(D) | 3,62137 | 9,06E-05 |
| C1GAS0 | Cystathionine gamma-synthase | UP (8S) | 3,604537 | 0,031634 |
| A0A0A0HTR8 | omega-amidase [EC:3.5.1.3] | UP (12S) | 3,526844 | 0,036284 |
| C1FYE6 | Glutaminase A | UP(D) | 3,433912 | 0,000121 |
| C1G020 | Glycine cleavage system P protein | UP(D) | 3,416136 | 5,67E-05 |
| C1GHU8 | 2-isopropylmalate synthase | UP(D) | 3,391257 | 9,61E-05 |
| C1GGZ4 | Phosphoserine transaminase | UP(D) | 3,384233 | 4,92E-05 |
| C1G765 | Alanine--glyoxylate transaminase | UP(D) | 3,339134 | 0,00031 |
| C1G8D6 | S-methyl-5'-thioadenosine phosphorylase | UP(D) | 3,318353 | 8,65E-05 |
| C1GMI2 | 4-hydroxyphenylpyruvate dioxygenase [EC:1.13.11.27] | UP(D) | 3,276808 | 0,000274 |
| C1GHM0 | Histidine biosynthesis trifunctional protein | UP (8S) | 3,268918 | 0,044375 |
| C1GK60 | Aromatic amino acid aminotransferase | UP (12S) | 3,262597 | 0,027969 |
| C1GLT7 | 5-methyltetrahydropteroyltriglutamate--homocysteine S-methyltransferase | UP(D) | 3,189006 | 4,11E-05 |
| C1G7F9 | Phospho-2-dehydro-3-deoxyheptonate aldolase | UP(D) | 3,188288 | 6,5E-05 |
| C1G7L2 | 3-isopropylmalate dehydratase [EC:4.2.1.33] | UP(D) | 3,188138 | 0,000188 |
| C1G8Z9 | Proline iminopeptidase | UP(D) | 3,179835 | 0,000123 |
| C1GLF6 | L-2-aminoadipate reductase [EC:1.2.1.95] | UP (12S) | 3,167476 | 0,025008 |
| C1G2Y9 | Argininosuccinate lyase | UP(D) | 3,114458 | 9,06E-05 |
| C1GM15 | O-acetylhomoserine (Thiol)-lyase | UP (12S) | 3,101635 | 0,025008 |
| C1G4M0 | Adenosylhomocysteinase | UP(D) | 3,086104 | 4,39E-05 |
| C1GBD8 | Succinyl-CoA:3-ketoacid-coenzyme A transferase | UP(D) | 3,081774 | 0,0002 |
| C1FZL3 | dipeptidyl aminopeptidase B [EC:3.4.14.-] | UP(D) | 3,072322 | 0,000123 |
| C1G3G3 | gamma-glutamyltranspeptidase / glutathione hydrolase [EC:2.3.2.2 3.4.19.13] | UP(D) | 2,990363 | 0,000499 |
| C1G5K1 | Cystathionine gamma-lyase | UP (8S) | 2,986016 | 0,022455 |
| C1GG82 | Glutamate decarboxylase | UP(D) | 2,912588 | 4,39E-05 |
| C1GLY5 | Aspartate-semialdehyde dehydrogenase | UP(D) | 2,746828 | 0,000328 |
| C1G3M0 | Glutamine synthetase | UP (8S) | 2,745705 | 0,026668 |
| C1GBT4 | Delta-1-pyrroline-5-carboxylate dehydrogenase A | UP(D) | 2,737999 | 4,39E-05 |
| C1G197 | Arginase | UP(D) | 2,727732 | 9,37E-05 |
| C1GIR0 | Pyrroline-5-carboxylate reductase | UP(D) | 2,693607 | 9,35E-05 |
| C1G792 | threonine dehydratase [EC:4.3.1.19] | UP (8S) | 2,628232 | 0,0352 |
| C1G7A3 | succinate-semialdehyde dehydrogenase / glutarate-semialdehyde dehydrogenase [EC:1.2.1.16 1.2.1.79 1.2.1.20] | UP (8S) | 2,588275 | 0,024733 |
| C1GCG4 | Glutamine synthetase | UP (12S) | 2,514859 | 0,025901 |
| C1GF52 | Aspartokinase | UP(D) | 2,461729 | 0,000241 |
| C1FYL2 | Argininosuccinate synthase | UP(D) | 2,398596 | 9,37E-05 |
| C1GDG5 | cystathionine beta-synthase [EC:4.2.1.22] | UP(D) | 2,37361 | 0,000138 |
| C1GC00 | Homoserine kinase | UP (12S) | 2,363345 | 0,033351 |
| C1GJD4 | Methylcrotonoyl-CoA carboxylase | UP (8S) | 2,315768 | 0,035838 |
| C1GD55 | 5-oxoprolinase (ATP-hydrolysing) [EC:3.5.2.9] | UP (8S) | 2,266957 | 0,033972 |
| C1G3B0 | Galactonate dehydratase | UP(D) | 2,218246 | 0,000121 |
| C1FYJ9 | Pentafunctional AROM polypeptide | UP (8S) | 2,20568 | 0,037983 |
| C1GMI0 | Homogentisate 1,2-dioxygenase | UP(D) | 2,067991 | 0,000408 |
| C1G388 | Aspartate aminotransferase | UP(D) | 2,036754 | 0,000403 |
| C1GBZ4 | NADP-specific glutamate dehydrogenase | UP (8S) | 1,9967 | 0,032085 |
| C1G989 | NAD-specific glutamate dehydrogenase | UP (8S) | 1,654924 | 0,042777 |
| **Lipid metabolism** | | |  |  |
| C1GDH4 | leukotriene-A4 hydrolase [EC:3.3.2.6] | UP(D) | 4,263176 | 0,000274 |
| C1GMW7 | Sphingolipid C9-methyltransferase A | UP (8S) | 3,572532 | 0,037729 |
| C1G736 | Acyl-coenzyme A oxidase | UP (8S) | 3,549005 | 0,046555 |
| C1GD39 | Ankyrin repeat protein nuc-2 | UP (12S) | 3,460394 | 0,025036 |
| C1G054 | Enoyl reductase (ER) domain-containing protein | UP (8S) | 3,424941 | 0,022455 |
| C1GBJ3 | ATP citrate synthase | UP (8S) | 3,126803 | 0,021452 |
| C1G0P4 | long-chain acyl-CoA synthetase [EC:6.2.1.3] | UP (12S) | 3,064416 | 0,032289 |
| C1G7I8 | Phosphatidic acid phosphatase type 2/haloperoxidase domain-containing protein | UP (8S) | 3,039503 | 0,038532 |
| C1G064 | fatty acid synthase subunit alpha, fungi type [EC:2.3.1.86] | UP (8S) | 2,628762 | 0,021657 |
| C1G065 | Fatty acid synthase beta subunit dehydratase | UP (8S) | 2,600071 | 0,021657 |
| C1GDJ1 | acetyl-CoA carboxylase / biotin carboxylase 1 [EC:6.4.1.2 6.3.4.14 2.1.3.15] | UP (8S) | 2,581906 | 0,024235 |
| C1GBJ2 | ATP citrate synthase | UP(D) | 2,453147 | 0,000166 |
| C1G121 | Galactokinase | UP (12S) | 1,724344 | 0,047864 |
| **Other metabolisms** | | |  |  |
| C1G421 | acetyl-CoA C-acetyltransferase | UP(D) | 6,076812 | 4,39E-05 |
| C1G839 | Aldehyde dehydrogenase domain-containing protein | UP (8S) | 5,781907 | 0,019701 |
| C1G3P8 | Methylmalonate-semialdehyde dehydrogenase (CoA acylating)[...] | UP (8S) | 5,466373 | 0,023829 |
| C1GI49 | Phosphatidylinositol 4-kinase | UP (8S) | 5,121642 | 0,044674 |
| C1G3Y2 | Alpha-1,6-mannosyltransferase subunit | UP (8S) | 4,92358 | 0,038532 |
| C1G215 | Phosphoglycolate phosphatase | UP (8S) | 4,909132 | 0,025741 |
| C1G7P5 | Galactose-1-phosphate uridylyltransferase | UP (12S) | 4,814447 | 0,038065 |
| C1GAY4 | peptide-methionine (S)-S-oxide reductase [EC:1.8.4.11] | UP (8S) | 4,707147 | 0,019701 |
| C1G167 | Riboflavin synthase, alpha subunit | UP (12S) | 4,694914 | 0,025036 |
| C1FZT6 | Gamma-butyrobetaine dioxygenase | UP(D) | 4,613136 | 6,85E-05 |
| C1FZG9 | AMP deaminase | UP (8S) | 4,534948 | 0,037294 |
| C1GK32 | Succinate-semialdehyde dehydrogenase | UP (8S) | 4,292799 | 0,045621 |
| C1GG60 | Phosphoribosylamine-glycine ligase | UP (12S) | 4,132568 | 0,04321 |
| C1G356 | Mannitol-1-phosphate 5-dehydrogenase | UP (12S) | 4,065633 | 0,026454 |
| C1G0Q3 | Dihydropteroate synthase | UP (12S) | 3,876472 | 0,048786 |
| C1GAM6 | UDP-N-acetylglucosamine pyrophosphorylase | UP (12S) | 3,836522 | 0,025008 |
| C1G8F8 | Ser/Thr protein phosphatase | UP(D) | 3,750471 | 0,000188 |
| C1GGQ7 | Dihydrolipoyl dehydrogenase | UP(D) | 3,669436 | 4,33E-05 |
| C1G7A4 | Alpha-galactosidase | UP(D) | 3,633348 | 0,000115 |
| C1GGE5 | acetyl-CoA C-acetyltransferase | UP(D) | 3,504626 | 3,89E-05 |
| C1GFU4 | GCS light chain | UP (8S) | 3,44275 | 0,038616 |
| C1G7R6 | metallopeptidase MepB [EC:3.4.24.-] | UP(D) | 3,437973 | 0,000307 |
| C1GD57 | Xaa-Pro dipeptidase [EC:3.4.13.9] | UP(D) | 3,42764 | 0,00012 |
| C1GDE1 | Serine hydroxymethyltransferase | UP(D) | 3,406268 | 0,000111 |
| C1G0A8 | FGGY-family pentulose kinase | UP (12S) | 3,376875 | 0,042742 |
| C1G280 | Adenosine kinase | UP (12S) | 3,37596 | 0,031427 |
| C1GC81 | Spermidine synthase | UP(D) | 3,375949 | 0,00011 |
| C1GCJ6 | 2-methylcitrate dehydratase | UP (12S) | 3,340075 | 0,028417 |
| C1G3A8 | Inosine-uridine preferring nucleoside hydrolase | UP (12S) | 3,312404 | 0,041282 |
| C1G0D2 | Xanthine phosphoribosyltransferase 1 | UP (12S) | 3,290465 | 0,033178 |
| A0A0A0HTY3 | 1,4-alpha-glucan branching enzyme [EC:2.4.1.18] | UP (12S) | 3,28858 | 0,031427 |
| C1G7X1 | S-(hydroxymethyl)glutathione dehydrogenase | UP (12S) | 3,284457 | 0,025901 |
| C1GGV9 | Aminopeptidase | UP(D) | 3,283653 | 0,000307 |
| C1GIF1 | Glycosyl hydrolase family 63 C-terminal domain-containing protein | UP (12S) | 3,281181 | 0,044079 |
| C1GCX5 | glycine hydroxymethyltransferase [EC:2.1.2.1] | UP (8S) | 3,116455 | 0,026668 |
| C1GK20 | D-xylose reductase [NAD(P)H] | UP(D) | 3,101768 | 0,000307 |
| C1G9K7 | phosphomannomutase [EC:5.4.2.8] | UP (12S) | 3,097256 | 0,025008 |
| C1GJZ9 | inosine-5'-monophosphate dehydrogenase | UP (8S) | 3,091429 | 0,021626 |
| C1FZ38 | Phospholipase | UP(D) | 3,081991 | 0,000121 |
| C1GEY4 | Xaa-Pro aminopeptidase [EC:3.4.11.9] | UP(D) | 3,030562 | 6,5E-05 |
| A0A0A0HR31 | Phosphoribosylaminoimidazole carboxylase | UP (8S) | 2,990958 | 0,019884 |
| C1G7L7 | Methylenetetrahydrofolate reductase | UP (8S) | 2,982698 | 0,03136 |
| C1G1J4 | Urea carboxylase | UP(D) | 2,887167 | 0,00037 |
| C1G8N6 | WD_REPEATS_REGION domain-containing protein | UP (8S) | 2,863348 | 0,021452 |
| C1GCG5 | Glucose 1-dehydrogenase | UP(D) | 2,855205 | 0,00018 |
| C1G2L8 | Delta-aminolevulinic acid dehydratase | UP (12S) | 2,839462 | 0,033966 |
| C1GCI7 | Isocitrate lyase | UP(D) | 2,824223 | 5,05E-05 |
| C1G9D5 | urease [EC:3.5.1.5] | UP (12S) | 2,757497 | 0,025901 |
| C1G164 | phosphoacetylglucosamine mutase [EC:5.4.2.3] | UP(D) | 2,721374 | 0,000109 |
| C1FYN6 | UDP-galactopyranose mutase | UP(D) | 2,691228 | 8,58E-05 |
| C1G1X9 | Alpha-1,4 glucan phosphorylase | UP (8S) | 2,665485 | 0,023614 |
| C1G7X3 | inositol-3-phosphate synthase | UP(D) | 2,622719 | 9,06E-05 |
| C1G018 | Choline-sulfatase | UP(D) | 2,616412 | 0,000295 |
| C1GJT8 | Nucleoside diphosphate kinase | UP (12S) | 2,583002 | 0,025655 |
| C1GML7 | Phosphoenolpyruvate carboxykinase (ATP) | UP(D) | 2,559044 | 0,000139 |
| C1GFB1 | Nicotinate-nucleotide pyrophosphorylase [carboxylating] | UP(D) | 2,542969 | 0,000114 |
| A0A0A0HT60 | DNA/pantothenate metabolism flavoprotein C-terminal domain-containing protein | UP(D) | 2,531657 | 0,000257 |
| C1GB27 | adenylosuccinate lyase [EC:4.3.2.2] | UP(D) | 2,480118 | 5,33E-05 |
| C1GA13 | Bifunctional purine biosynthesis protein ADE17 | UP(D) | 2,451998 | 9,12E-05 |
| C1G1K5 | Acyl-CoA thioesterase II | UP (12S) | 2,439065 | 0,025901 |
| C1GC82 | Transketolase | UP(D) | 2,400393 | 0,000208 |
| C1GBS0 | carbamoyl-phosphate synthase / aspartate carbamoyltransferase [EC:6.3.5.5 2.1.3.2] | UP (8S) | 2,38421 | 0,028431 |
| C1GA17 | Pyruvate carboxylase | UP (8S) | 2,270743 | 0,023878 |
| C1GAT8 | UTP--glucose-1-phosphate uridylyltransferase [EC:2.7.7.9] | UP (8S) | 2,244107 | 0,027348 |
| C1GEU2 | Glycogen [starch] synthase | UP (8S) | 2,130207 | 0,035027 |
| C1GEY6 | pyridoxal 5'-phosphate synthase pdxS subunit [EC:4.3.3.6] | UP (12S) | 2,110704 | 0,032289 |
| C1GHS5 | Amidase | UP (12S) | 2,001677 | 0,040086 |
| C1GB99 | Trehalose-6-phosphate synthase | UP (8S) | 1,819786 | 0,039312 |
| C1G411 | Acetyl-coenzyme A synthetase | UP(D) | 1,743178 | 0,000307 |
| **Cell cycle** | |  |  |  |
| C1GL30 | Velvet domain-containing protein | UP (8S) | 5,187395 | 0,049524 |
| C1G681 | Reduced viability upon starvation protein | UP (8S) | 5,039071 | 0,020855 |
| C1FYS5 | Microtubule-associated protein RP/EB family member 3 | UP(D) | 4,63316 | 6,72E-05 |
| C1FZ18 | DNA polymerase delta subunit 3 | UP(D) | 4,293737 | 0,000105 |
| C1G7R4 | Myosin regulatory light chain cdc4 | UP(D) | 3,978842 | 5,33E-05 |
| C1G0X2 | SCF E3 ubiquitin ligase complex F-box protein grrA | UP (8S) | 3,93618 | 0,026402 |
| C1G857 | Cohesin complex subunit SA-1/2 | UP (8S) | 3,849044 | 0,038532 |
| C1G5N0 | Casein kinase II subunit alpha | UP(D) | 3,547176 | 4,39E-05 |
| A0A0A0HUN4 | Mitotic checkpoint protein BUB3 | UP(D) | 3,294945 | 6,61E-05 |
| C1GEQ3 | NAP family protein | UP(D) | 3,209793 | 0,000138 |
| C1GG95 | Pro-apoptotic serine protease NMA111 | UP (8S) | 3,09055 | 0,029186 |
| C1G389 | serine/threonine-protein phosphatase 2A regulatory subunit A | UP (12S) | 2,743625 | 0,033901 |
| C1G8Q1 | Cyclin-dependent kinase 1 | UP (8S) | 2,537669 | 0,037729 |
| C1G5B1 | DNA replication licensing factor MCM2 | UP (8S) | 2,211837 | 0,045673 |
| C1GBH3 | Serine/threonine-protein phosphatase PP2A catalytic subunit | UP(D) | 2,019703 | 0,000123 |
| **Signal transduction** | |  |  |  |
| C1G501 | Calmodulin | UP (8S) | 7,115894 | 0,033522 |
| C1G887 | GTP-binding protein ryh1 | UP (8S) | 5,255056 | 0,02906 |
| C1GFR7 | GTPase activating protein | UP (8S) | 5,13472 | 0,021452 |
| C1GKC8 | Ras GTPase-activating protein-binding protein 2 | UP (8S) | 4,596739 | 0,020855 |
| C1GE97 | SEC7 domain-containing protein | UP(D) | 4,478274 | 4,39E-05 |
| C1GJ85 | DH domain-containing protein | UP (8S) | 3,807518 | 0,020855 |
| C1GGW3 | Glucose repression regulatory protein TUP1 | UP (8S) | 3,664806 | 0,020855 |
| C1GJJ1 | Serine/threonine-protein phosphatase | UP (12S) | 3,052597 | 0,025008 |
| C1G2S7 | Rab GDP dissociation inhibitor | UP(D) | 2,711735 | 0,000626 |
| C1FYR1 | Rab family, other | UP (8S) | 2,64551 | 0,040422 |
| A0A0A0HVP8 | Mitogen-activated protein kinase | UP (12S) | 2,554857 | 0,041366 |
| C1GG36 | Calcineurin subunit B | UP(D) | 2,285403 | 9,06E-05 |
| **Cytoskeleton** | |  |  |  |
| C1GJ47 | Alpha-actinin | UP(D) | 6,295237 | 4,39E-05 |
| C1GFT4 | Cytoskeleton assembly control protein | UP (8S) | 4,252275 | 0,019701 |
| C1G1V9 | Actin-related protein 2 | UP (8S) | 3,047724 | 0,022455 |
| C1GJ13 | coronin-1B/1C/6 | UP(D) | 2,812665 | 0,000152 |
| C1GKE7 | Arp2/3 complex 34 kDa subunit | UP (8S) | 2,53266 | 0,023878 |
| C1G9M3 | Actin-related protein 2/3 complex subunit | UP (8S) | 2,464058 | 0,028369 |
| A0A0A0HRG7 | Actin | UP(D) | 2,416536 | 0,001627 |
| C1G0N2 | Actin cytoskeleton protein (VIP1) | UP(D) | 2,35749 | 9,12E-05 |
| C1GM22;C1G3F2 | Tubulin alpha chain | UP (12S) | 2,033731 | 0,038172 |
| C1G3I9 | Actin-like protein | UP (12S) | 1,676152 | 0,049928 |
| **Transport** | |  |  |  |
| C1GFU3 | AP complex subunit sigma | UP (8S) | 5,662221 | 0,029047 |
| A0A0A0HUB3 | Oxysterol binding protein (Osh1) | UP (8S) | 5,287759 | 0,019701 |
| C1GGX1 | ABC transporter domain-containing protein | UP(D) | 5,18115 | 3,95E-05 |
| C1FZK1 | Ran GTPase-activating protein | UP(D) | 5,0913 | 0,000269 |
| A0A0A0HVG2 | Importin N-terminal domain-containing protein | UP(D) | 4,311295 | 9,12E-05 |
| C1GM90 | Phosphatidylinositol-phosphatidylcholine transfer protein | UP (12S) | 4,194224 | 0,048674 |
| C1GLV1 | GTP-binding protein ypt1 | UP (8S) | 4,112666 | 0,023769 |
| C1G2A4 | Sodium/hydrogen exchanger | UP (8S) | 3,992395 | 0,020855 |
| C1GCD5 | Nascent polypeptide-associated complex subunit beta | UP (12S) | 3,660537 | 0,028963 |
| C1GIR1 | Chloride channel protein | UP (8S) | 3,649266 | 0,022455 |
| C1GLJ9 | Importin N-terminal domain-containing protein | UP (12S) | 3,56403 | 0,025036 |
| C1G6Y4 | carnitine O-acetyltransferase [EC:2.3.1.7] | UP (8S) | 3,561004 | 0,029126 |
| C1GG46 | Hypercellular protein HypA | UP (8S) | 3,557669 | 0,02279 |
| A0A0A0HVN6 | Microtubule binding protein HOOK3 | UP (8S) | 3,523648 | 0,020879 |
| C1GG11 | Vacuolar protein sorting-associated protein | UP(D) | 3,475178 | 0,000297 |
| C1GHB2 | Importin N-terminal domain-containing protein | UP (12S) | 3,359892 | 0,025655 |
| C1G3Q1 | Sorting nexin 3 | UP (8S) | 3,269097 | 0,020855 |
| C1G3N9 | Sorting nexin-4 | UP (8S) | 3,180209 | 0,049285 |
| C1G555 | Importin N-terminal domain-containing protein | UP (12S) | 3,084482 | 0,025901 |
| C1GA14 | Clathrin heavy chain | UP (8S) | 3,052593 | 0,021908 |
| C1GDU8 | Chloride channel protein | UP(D) | 3,043812 | 6,78E-05 |
| C1GK28 | Importin N-terminal domain-containing protein | UP (8S) | 3,01078 | 0,037729 |
| C1GDL1 | Phospholipid-transporting ATPase | UP(D) | 2,994825 | 0,00019 |
| C1G4D4 | Vacuolar protein sorting-associated protein 26 | UP (8S) | 2,989498 | 0,020855 |
| C1GEQ4 | t-SNARE coiled-coil homology domain-containing protein | UP(D) | 2,987106 | 4,39E-05 |
| A0A0A0HXV1 | AP complex subunit beta | UP (8S) | 2,985063 | 0,031212 |
| C1G2M4 | Protein YIP | UP (8S) | 2,945987 | 0,021908 |
| C1G0N4 | Importin N-terminal domain-containing protein | UP(D) | 2,913918 | 9,12E-05 |
| C1GJS2 | Phosphatidylinositol transfer protein SFH5 | UP(D) | 2,90578 | 0,000328 |
| C1G2L0 | Importin N-terminal domain-containing protein | UP(D) | 2,884325 | 0,0007 |
| C1G182 | ATPase GET3 | UP(D) | 2,852553 | 9,35E-05 |
| C1G112 | Vacuolar protein sorting-associated protein 35 | UP (8S) | 2,706887 | 0,025544 |
| C1GLB9 | BET3 family protein | UP (8S) | 2,683259 | 0,048435 |
| C1GHE5 | importin subunit beta-1 | UP (12S) | 2,638573 | 0,031427 |
| C1GJN2 | AP-1 complex subunit mu | UP (8S) | 2,599376 | 0,042697 |
| C1FZ88 | Importin subunit alpha | UP(D) | 2,524766 | 8,58E-05 |
| C1GBG4 | coatomer subunit gamma | UP (8S) | 2,520392 | 0,038616 |
| C1GEK5 | Sorting nexin MVP1 | UP (12S) | 2,471847 | 0,041282 |
| C1GGB6 | Transmembrane 9 superfamily member | UP(D) | 2,414798 | 0,000156 |
| C1G0X9 | AP-1 complex subunit gamma | UP (8S) | 2,393857 | 0,042777 |
| C1GBM3 | Synaptobrevin homolog YKT6 | UP (8S) | 2,386595 | 0,048303 |
| C1G8G0 | importin-4 | UP (12S) | 2,363398 | 0,028744 |
| C1G3X8 | t-SNARE coiled-coil homology domain-containing protein | UP (8S) | 2,322506 | 0,026402 |
| C1GM08 | GTP-binding protein ypt3 | UP (12S) | 2,166641 | 0,03073 |
| C1G1M0 | Autophagy-related protein 27 | UP (12S) | 2,139261 | 0,0458 |
| C1GE52 | actin-related protein 3 | UP (8S) | 2,084922 | 0,037729 |
| C1G9U1 | COPII-coated vesicle component Erv46 | UP (8S) | 1,992882 | 0,034376 |
| C1G3M9 | Golgi apparatus membrane protein TVP2 | UP(D) | 1,850829 | 0,000356 |
| C1GCT8 | GTP-binding nuclear protein | UP (12S) | 1,842541 | 0,046914 |
| **Others** |  |  |  |  |
| C1GA81 | Aspartyl aminopeptidase | UP(D) | 8,596116 | 9,61E-05 |
| C1GFV7 | BAR adaptor protein RVS167 | UP (8S) | 7,524867 | 0,023829 |
| C1G0V6 | Protoglobin domain-containing protein | UP (12S) | 6,042788 | 0,037043 |
| C1GK88 | Carbonic anhydrase | UP(D) | 5,9348 | 5E-05 |
| C1G4I6 | Protein phosphatase | UP (8S) | 5,255486 | 0,03496 |
| C1GDM0 | Isochorismatase domain-containing protein | UP(D) | 4,819232 | 4,39E-05 |
| C1GGC2 | Cysteine proteinase 1, mitochondrial | UP (8S) | 4,51707 | 0,019701 |
| C1G9M7 | 30 kDa heat shock protein | UP(D) | 4,261656 | 9,12E-05 |
| C1GG52 | Lysine decarboxylase-like protein | UP (12S) | 4,094209 | 0,028257 |
| C1G5L8 | Pirin | UP (12S) | 4,035001 | 0,025008 |
| C1G9U6 | Uroporphyrinogen decarboxylase | UP (12S) | 4,02628 | 0,027518 |
| C1GLZ5 | Glycos_transf_1 domain-containing protein | UP(D) | 4,006454 | 3,44E-05 |
| C1GC65 | Glutathione peroxidase | UP(D) | 3,822248 | 3,44E-05 |
| C1FYY4 | AMP-activated protein kinase glycogen-binding domain-containing protein | UP(D) | 3,762252 | 0,000276 |
| C1G8J4 | Beta-lactamase domain-containing protein | UP (12S) | 3,734392 | 0,027951 |
| C1GGK1 | Chitin synthase | UP(D) | 3,727287 | 4,11E-05 |
| C1GJJ9 | Sorbitol utilization protein SOU2 | UP(D) | 3,717658 | 5,33E-05 |
| C1G217 | Nudix hydrolase domain-containing protein | UP(D) | 3,645371 | 0,000274 |
| C1G8E0 | M protein repeat protein | UP(D) | 3,600922 | 0,000195 |
| C1GEU7 | Farnesyl pyrophosphate synthetase | UP (12S) | 3,395626 | 0,028963 |
| C1GA02 | Zinc metalloprotease | UP(D) | 3,309445 | 0,000131 |
| C1GGY0 | Phenol 2-monooxygenase | UP(D) | 3,264951 | 9,12E-05 |
| C1G5J3 | ATP-dependent (S)-NAD(P)H-hydrate dehydratase | UP (12S) | 3,122294 | 0,025901 |
| C1GGQ3 | Formamidase | UP (12S) | 3,072688 | 0,025901 |
| C1GF19 | Lactonohydrolase | UP(D) | 2,911244 | 8,58E-05 |
| C1G5H5 | Bromo domain-containing protein | UP (8S) | 2,879184 | 0,03899 |
| C1FZS1 | Fe2OG dioxygenase domain-containing protein | UP (12S) | 2,603499 | 0,025655 |
| C1G3H0 | Short chain dehydrogenase/reductase family | UP (12S) | 2,532908 | 0,025901 |
| C1GHK4 | Betaine aldehyde dehydrogenase | UP(D) | 2,475961 | 0,00014 |
| C1GKQ4 | Chitin synthase | UP (8S) | 1,937166 | 0,035838 |
| C1GBT0 | Aldehyde dehydrogenase | UP(D) | 1,852013 | 0,0002 |
| C1GKQ6 | Chitin synthase | UP (8S) | 1,77528 | 0,045621 |
| **No annotation found** | | |  |  |
| C1GJH8 | NA | UP (8S) | 7,281491 | 0,023319 |
| C1G726 | ThiJ/PfpI family protein | UP (12S) | 6,673461 | 0,031613 |
| C1G760 | GPI anchored serine-threonine rich protein | UP (8S) | 6,450801 | 0,026668 |
| C1G8A9 | HET-C domain-containing protein HetC | UP (12S) | 6,388762 | 0,041723 |
| C1GEG2 | Integral membrane protein | UP (8S) | 5,592156 | 0,019701 |
| C1GFJ7 | NIPSNAP domain-containing protein | UP(D) | 5,358629 | 8,82E-05 |
| C1G0R9 | PBP domain-containing protein | UP (8S) | 5,302865 | 0,037294 |
| C1G6Q5 | GOLD domain-containing protein | UP (8S) | 5,135181 | 0,037729 |
| A0A0A0HT22 | AMPK1_CBM domain-containing protein | UP (8S) | 4,71295 | 0,026402 |
| C1GJT4 |  | UP (12S) | 4,471242 | 0,047992 |
| C1GG74 |  | UP (12S) | 4,190288 | 0,043268 |
| C1GM11 |  | UP (8S) | 4,180892 | 0,019701 |
| C1GCP8 | Uncharacterized protein | UP (12S) | 4,008724 | 0,044035 |
| C1GL73 |  | UP (8S) | 4,003707 | 0,037729 |
| C1GKX2 |  | UP(D) | 3,977313 | 5,67E-05 |
| C1GB68 | HIT domain-containing protein | UP(D) | 3,88923 | 3,89E-05 |
| C1G1D4 | Hva1_TUDOR domain-containing protein | UP (8S) | 3,650764 | 0,037729 |
| C1FYH3 | NA | UP (8S) | 3,639481 | 0,020855 |
| C1GLK2 | UPF0662 protein | UP (12S) | 3,57271 | 0,025036 |
| C1GHP7 | Uncharacterized protein | UP(D) | 3,430084 | 6,72E-05 |
| C1GBH2 |  | UP (8S) | 3,355685 | 0,044375 |
| C1G1R0 |  | UP (8S) | 3,304327 | 0,045621 |
| C1G3Z3 | CAP10 domain-containing protein | UP(D) | 3,256972 | 6,61E-05 |
| C1G1S6 |  | UP(D) | 3,251665 | 0,000928 |
| C1GBN1 | CS domain-containing protein | UP(D) | 3,239087 | 0,000135 |
| C1FYP5 |  | UP(D) | 3,162042 | 8,65E-05 |
| C1G712 | PH domain-containing protein | UP(D) | 3,146066 | 0,00018 |
| A0A0A0HUC7 | High-temperature-induced dauer-formation protein | UP (8S) | 3,087212 | 0,026402 |
| C1FYX7 | DM2 domain-containing protein | UP(D) | 3,034984 | 0,000188 |
| C1G1V7 | NADP-dependent oxidoreductase domain-containing protein | UP(D) | 3,014112 | 5,33E-05 |
| C1GAH4 |  | UP(D) | 2,880297 | 0,000111 |
| C1G4K3 | 4F5 domain-containing protein | UP (8S) | 2,851154 | 0,02906 |
| C1G1L3 | Hsp70-like protein | UP (8S) | 2,7821 | 0,021452 |
| C1G5X0 | Carrier domain-containing protein | UP(D) | 2,762938 | 0,000121 |
| C1G647 | NA | UP(D) | 2,520252 | 6,5E-05 |
| C1GE44 |  | UP(D) | 2,40046 | 8,58E-05 |
| C1GN52 | GPR/FUN34 family protein | UP (12S) | 2,166027 | 0,0458 |
| C1G126 | UPF0132 domain-containing protein | UP (8S) | 2,112519 | 0,02906 |
