# Supplementary material for: Extracellular vesicles from virulent P. brasiliensis induce TLR4 and dectin-1 expression in innate cells and promote enhanced Th1/Th17 response
Source: Virulence. 2024 Mar 21;15(1):2329573. doi: 10.1080/21505594.2024.2329573 (PMC10962619; doi:10.1080/21505594.2024.2329573)
Supplement: Supplemental Material [file KVIR_A_2329573_SM2101.zip › Supplementary Table 2.docx]

**Supplementary Table 2.**

| **Acession number** | **Protein** |
| --- | --- |
| **Virulence factor** | |
| C1G0T3 | Glycosyl transferase CAP10 domain-containing protein |
| **Gene/protein regulation** | |
| A0A0A0HXB4 | Leucine carboxyl methyltransferase 1 |
| C1FZ58 | Pseudouridine synthase RsuA/RluA-like domain-containing protein |
| C1FZJ4 | Transcription elongation factor S-II |
| C1FZQ1 | Transcriptional activator hap2 |
| C1FZT3 | Exonuclease domain-containing protein |
| C1G015 | Pre-mRNA-splicing factor slt11 |
| C1G030 | Transcription factor CBF/NF-Y/archaeal histone domain-containing protein |
| C1G136 | Pre-RNA splicing factor Srp2, variant 2 |
| C1G251 | Probable ubiquitin thioesterase DG1039 |
| C1G2C1 | Translation initiation factor eIF-2B subunit beta |
| C1G2I3 | Probable cytosolic iron-sulfur protein assembly protein 1 |
| C1G2Y0 | Methionine aminopeptidase |
| C1G2Z4 | U6 snRNA-associated Sm-like protein LSm6 |
| C1G3W8 | Geranylgeranyl transferase type-2 subunit alpha |
| C1G4J4 | Casein kinase II subunit beta |
| C1G4K2 | ATP-dependent RNA helicase |
| C1G4Q0 | Deacetylase sirtuin-type domain-containing protein |
| C1G4Q1 | Transcription factor CBF/NF-Y/archaeal histone domain-containing protein |
| C1G505 | CID domain-containing protein |
| C1G573 | rRNA biogenesis protein RRP5 |
| C1G582 | DnaJ domain-containing protein |
| C1G5E9 | Peptidylprolyl isomerase |
| C1G654 | THO complex subunit 2 |
| C1G6J2 | RNase III domain-containing protein |
| C1G6M0 | U6 snRNA-associated Sm-like protein LSm8 |
| C1G763 | RNA-binding domain-containing protein |
| C1G8X9 | Peptidylprolyl isomerase |
| C1G9D3 | HMG box domain-containing protein |
| C1G9Y0 | UBX domain-containing protein |
| C1GA39 | mitochondrial presequence protease |
| C1GAF3 | Superkiller protein 3 |
| C1GAF6 | mitochondrial FAD-linked sulfhydryl oxidase [EC:1.8.3.2] |
| C1GAG0 | tRNA (Guanine9-N1)-methyltransferase |
| C1GAG1 | tRNA (guanine(9)-N1)-methyltransferase |
| C1GAR5 | Alpha 1,2-mannosyltransferase |
| C1GAV8 | MYB DNA-binding domain-containing protein |
| C1GB00 | RRM domain-containing protein |
| C1GBF7 | ATP-dependent DNA helicase II subunit 2 |
| C1GBK4 | Ubiquitin-activating enzyme E1-like |
| C1GD00 | Exosome complex exonuclease RRP40 |
| C1GEE1 | XPG N-terminal domain-containing protein |
| C1GEF6 | SRP9-21 domain-containing protein |
| C1GER3 | C6 finger domain transcription factor nscR |
| C1GF79 | Ubiquitinyl hydrolase 1 |
| C1GFC3 | Casein kinase substrate phosphoprotein PP28 domain-containing protein |
| C1GGP9 | General negative regulator of transcription subunit |
| C1GGQ0 | D-aminoacyl-tRNA deacylase |
| C1GH73 | tRNA-dihydrouridine(47) synthase [NAD(P)(+)] |
| C1GHD0 | RRM domain-containing protein |
| C1GIA6 | GrpE protein homolog |
| C1GIQ1 | Ribosomal protein S10 domain-containing protein |
| C1GIR7 | Mitochondrial import inner membrane translocase subunit |
| C1GIU8 | U6 snRNA-associated Sm-like protein LSm6 |
| C1GJU9 | Histone demethylase JARID1 |
| C1GKV5 | MADS-box domain-containing protein |
| C1GL96 | HECT-type E3 ubiquitin transferase |
| C1GLR4 | KH domain-containing protein |
| C1GMD2 | Vacuolar protein sorting-associated protein 74 |
| C1GMJ8 | T-complex protein 1 subunit epsilon |
| C1GMU8 | Eukaryotic translation initiation factor 4E-1 |
| **Energy metabolism** | |
| C1FZL2 | Succinate dehydrogenase [ubiquinone] flavoprotein subunit, mitochondrial[...] |
| C1GAG2 | NADH dehydrogenase [ubiquinone] 1 alpha subcomplex subunit 13 |
| C1GHV7 | V-type proton ATPase subunit |
| **Amino acid metabolism** | |
| A0A0A0HQD9 | Copper transport protein 86 |
| A0A0A0HS25 | DAO domain-containing protein |
| A0A0A0HWJ2 | Amidase 1 |
| C1G025 | Aromatic-L-amino-acid decarboxylase |
| C1G1L5 | N2227 domain-containing protein |
| C1G4C3 | Anthranilate phosphoribosyltransferase |
| C1G553 | GST N-terminal domain-containing protein |
| C1G6Z1 | D-serine dehydratase-like domain-containing protein |
| C1G7B2 | Peptide hydrolase |
| C1G900 | Tyrosine decarboxylase |
| C1GF82 | Cystathionine gamma-synthase |
| C1GFM4 | Phosphoserine phosphatase |
| C1GGZ6 | Homoaconitase, mitochondrial |
| C1GL38 | Branched-chain amino acid aminotransferase |
| C1GLQ9 | Ornithine carbamoyltransferase, mitochondrial |
| **Lipid metabolisms** | |
| C1G1D8 | Oxysterol binding protein |
| C1GCU5 | SCP2 domain-containing protein |
| C1GF96 | Oxysterol-binding protein |
| C1GFA5 | Phospholipase D |
| C1GFW9 | Palmitoyl-protein thioesterase 1 |
| C1GHL5 | LNS2/PITP domain-containing protein |
| C1GLD2 | LEM3 family/CDC50 family protein |
| **Other metabolisms** | |
| A0A0A0HU08 | Dihydroneopterin aldolase |
| A0A0A0HUH4 | Thiamine diphosphokinase |
| A0A0A0HUY0 | GTP cyclohydrolase N-terminal domain-containing protein |
| A0A0A0HWA2 | Protein-ribulosamine 3-kinase |
| C1G3G8 | Cytosolic neutral trehalase |
| C1G423 | Arylamine N-acetyltransferase |
| C1G5I8 | carbamoyl-phosphate synthase large subunit [EC:6.3.5.5] |
| C1GB66 | Allantoate amidohydrolase |
| C1GBL6 | Chitin deacetylase |
| C1GDX6 | Pyridox_oxase_2 domain-containing protein |
| C1GE78 | Porphobilinogen deaminase |
| C1GFB5 | Aldose 1-epimerase |
| C1GFL6 | Precorrin-2 dehydrogenase |
| C1GHU5 | N-acetyltransferase domain-containing protein |
| C1GL99 | Alcohol dehydrogenase iron-type/glycerol dehydrogenase GldA domain-containing protein |
| **Siderophore related** | |
| C1G110 | Fusarinine C esterase sidJ |
| **Cell cycle** | |
| C1G177 | Spindle pole body component |
| C1GBL1 | Zinc finger protein zpr1 |
| C1GLH3 | Autophagy-related protein |
| **Cytoskeleton** | |
| C1G3P6 | Dynactin subunit 2 |
| C1GE05 | ADF-H domain-containing protein |
| **Signal transduction** | |
| A0A0A0HT87 | Rab proteins geranylgeranyltransferase |
| C1FZM8 | Rho-GAP domain-containing protein |
| C1G6I9 | Chimerin |
| **Transport** | |
| C1FYF2 | Peroxin-19 |
| C1FYK9 | MHD domain-containing protein |
| C1FZ16 | 60S ribosomal export protein NMD3 |
| C1FZN2 | Trafficking protein particle complex subunit |
| C1G226 | Autophagy-related protein 3 |
| C1G4G1 | Protein transport protein SFT2 |
| C1G6L8 | COPII-coated vesicle protein SurF4/Erv29 |
| C1GB34 | Protein MLP1 homolog |
| C1GBR3 | Kinesin motor domain-containing protein |
| C1GGX4 | DUF410 domain-containing protein |
| C1GHB9 | Coatomer subunit delta |
| C1GMA2 | Vacuolar-sorting protein snf7 |
| C1GMG5 | VHS domain-containing protein |
| **Others** |  |
| C1FZ10 | L-type lectin-like domain-containing protein |
| C1FZR6 | Ectonucleotide pyrophosphatase/phosphodiesterase family member 1/3 |
| C1G113 | Stress response protein NST1 |
| C1GBW5 | Chorismate synthase |
| C1GDY8 | Nudix hydrolase domain-containing protein |
| C1GED3 | Glycerate-and formate-dehydrogenase |
| C1GHW9 | Dihydroxyacetone kinase |
| C1GI55 | Mitochondrial fission 1 protein |
| C1GIQ3 | Two-component system protein A |
| **No annotation found** | |
| C1G031 | Short-chain dehydrogenase |
| C1G1H9 | Lgl_C domain-containing protein |
| C1G2Q7 | Uncharacterized protein |
| C1G2U5 | Uncharacterized protein |
| C1G3W9 | GTPase activating protein |
| C1G4G5 | ANK_REP_REGION domain-containing protein |
| C1G4L4 | VWFA domain-containing protein |
| C1G6P0 | CORD and CS domain-containing protein |
| C1G6X6 | DUF255 domain-containing protein |
| C1G8M6 | Uncharacterized protein |
| C1G8X5 | Uncharacterized protein |
| C1G9J8 | Uncharacterized protein |
| C1GB06 |  |
| C1GBA9 | YbgI/family dinuclear metal center protein |
| C1GBH1 |  |
| C1GBH7 | Tetratricopeptide repeat protein 1 |
| C1GBQ4 | Glyco_hydro_cc domain-containing protein |
| C1GE73 | Uncharacterized protein |
| C1GEP1 | LCCL domain-containing protein |
| C1GEX2 | VLRF1/Vms1 domain-containing protein |
| C1GFI5 | Uncharacterized protein |
| C1GHF4 |  |
| C1GI46 | Uncharacterized protein |
| C1GJI5 | DUF4704 domain-containing protein |
| C1GJP1 | ANK_REP_REGION domain-containing protein |
| C1GKC4 | Uncharacterized protein |
| C1GKI3 | Fungal-type protein kinase domain-containing protein |
