# Supplementary material for: Extracellular vesicles from virulent P. brasiliensis induce TLR4 and dectin-1 expression in innate cells and promote enhanced Th1/Th17 response
Source: Virulence. 2024 Mar 21;15(1):2329573. doi: 10.1080/21505594.2024.2329573 (PMC10962619; doi:10.1080/21505594.2024.2329573)
Supplement: Supplemental Material [file KVIR_A_2329573_SM2101.zip › Supplementary Table 3.docx]

**Supplementary Table 3.**

| **Acession number** | **Protein** |
| --- | --- |
| **Virulence factor** | |
| C1G532 | Nitroreductase domain-containing protein |
| C1GMI9 | Bys1 family protein |
| **Gene/protein regulation** | |
| A0A0A0HTL6 | Exoribonuclease phosphorolytic domain-containing protein |
| A0A0A0HUW4 | Non-specific serine/threonine protein kinase |
| C1FYP7 | Histone transcription regulator 3 homolog |
| C1FYV9 | Vacuolar assembly protein |
| C1G7A2 | Fungal-type protein kinase domain-containing protein |
| C1G0V1 | TATA-binding protein-associated factor mot1 |
| C1G254 | Pre-rRNA-processing protein RIX1 |
| C1G374 | CMGC/DYRK/YAK protein kinase |
| C1G3A3 | Ribosome-interacting GTPase 1 |
| C1G702 | Cell differentiation protein rcd1 |
| C1G8T4 | DRIM domain-containing protein |
| C1G9A6 | Mediator of RNA polymerase II transcription subunit 14 |
| C1GD85 | Polyadenylation factor subunit 2 |
| C1GE49 | 37S ribosomal protein S5 |
| C1GE75 | Non-specific serine/threonine protein kinase |
| C1GH40 | Endoribonuclease ysh1 |
| C1GI13 | Ribosomal protein/NADH dehydrogenase domain-containing protein |
| C1GIX3 | AAA+ ATPase domain-containing protein |
| C1GK54 | Pre-mRNA-splicing factor ATP-dependent RNA helicase PRP16 |
| C1GLK3 | 60S acidic ribosomal protein P1 |
| C1GLK8 | Transcription factor 25 |
| C1GMR0 | Mitochondrial ribosomal protein DAP3 |
| C1GMT5 | Isoleucine--tRNA ligase |
| C1GN14 | PAN2-PAN3 deadenylation complex catalytic subunit PAN2 |
| **Amino acid metabolisms** | |
| C1G6H7 | FAD dependent oxidoreductase domain-containing protein |
| **Other metabolisms** | |
| A0A0A0HQV2 | NAD(P)H-hydrate epimerase [EC:5.1.99.6] |
| C1G956 | Carbohydrate kinase PfkB domain-containing protein |
| **Transport** | |
| C1G3V8 | Exocyst complex component Sec6 |
| C1G6G7 | VPS9 domain-containing protein |
| C1G812 | Sorting nexin-3 |
| C1GDT0 | Polyadenylation factor subunit 2 |
| C1GFG2 | Nucleoporin NUP37 |
| **Others** |  |
| A0A0A0HRY5 | Tr-type G domain-containing protein |
| C1G3R3 | Calcineurin-like phosphoesterase domain-containing protein |
