# Supplementary figures and images for: Extracellular vesicles from virulent P. brasiliensis induce TLR4 and dectin-1 expression in innate cells and promote enhanced Th1/Th17 response
Source: Virulence. 2024 Mar 21;15(1):2329573. doi: 10.1080/21505594.2024.2329573 (PMC10962619; doi:10.1080/21505594.2024.2329573)

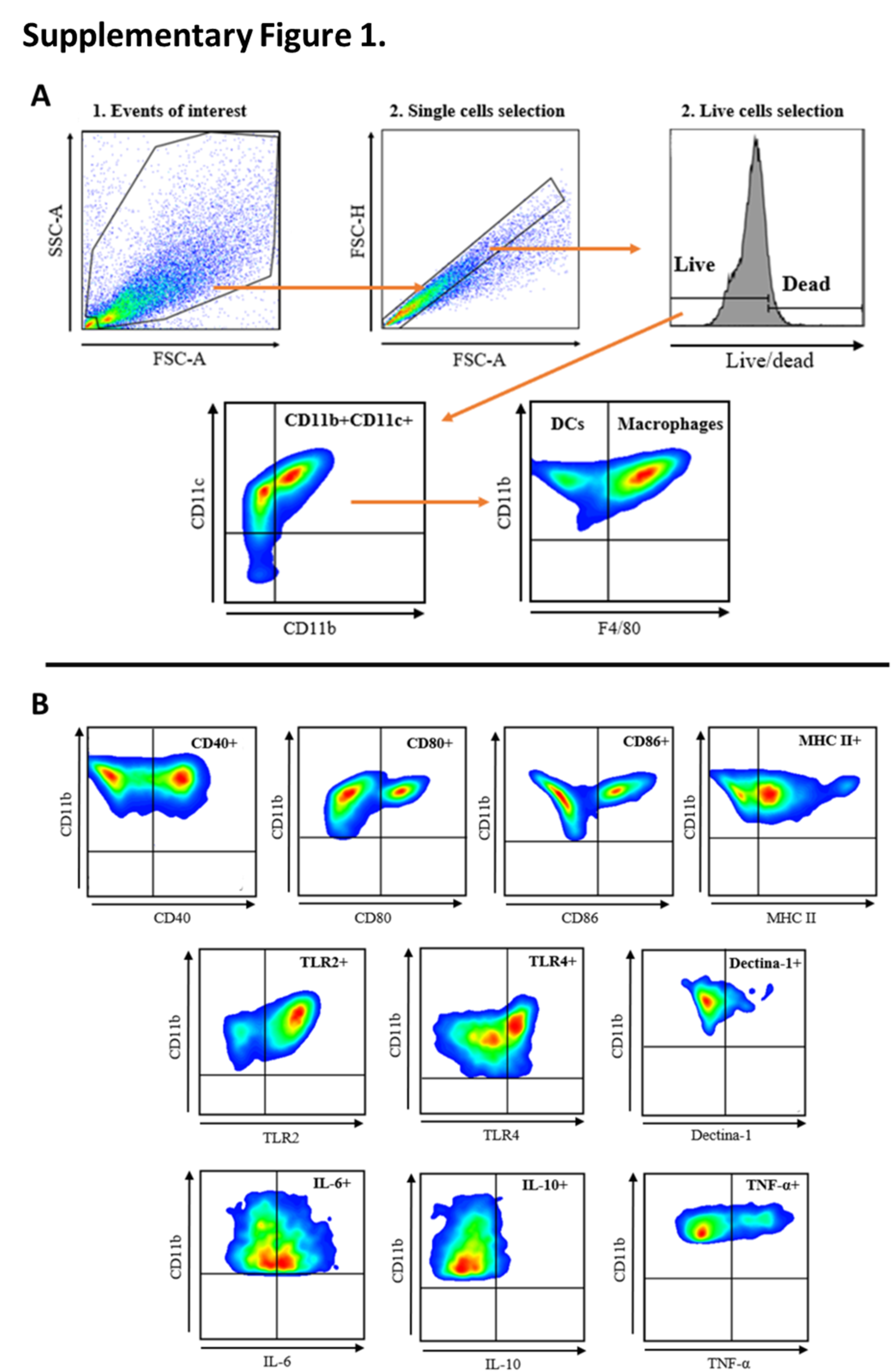

Supplement: Supplemental Material [file KVIR_A_2329573_SM2101.zip › Supplementary Figure 1.tiff]

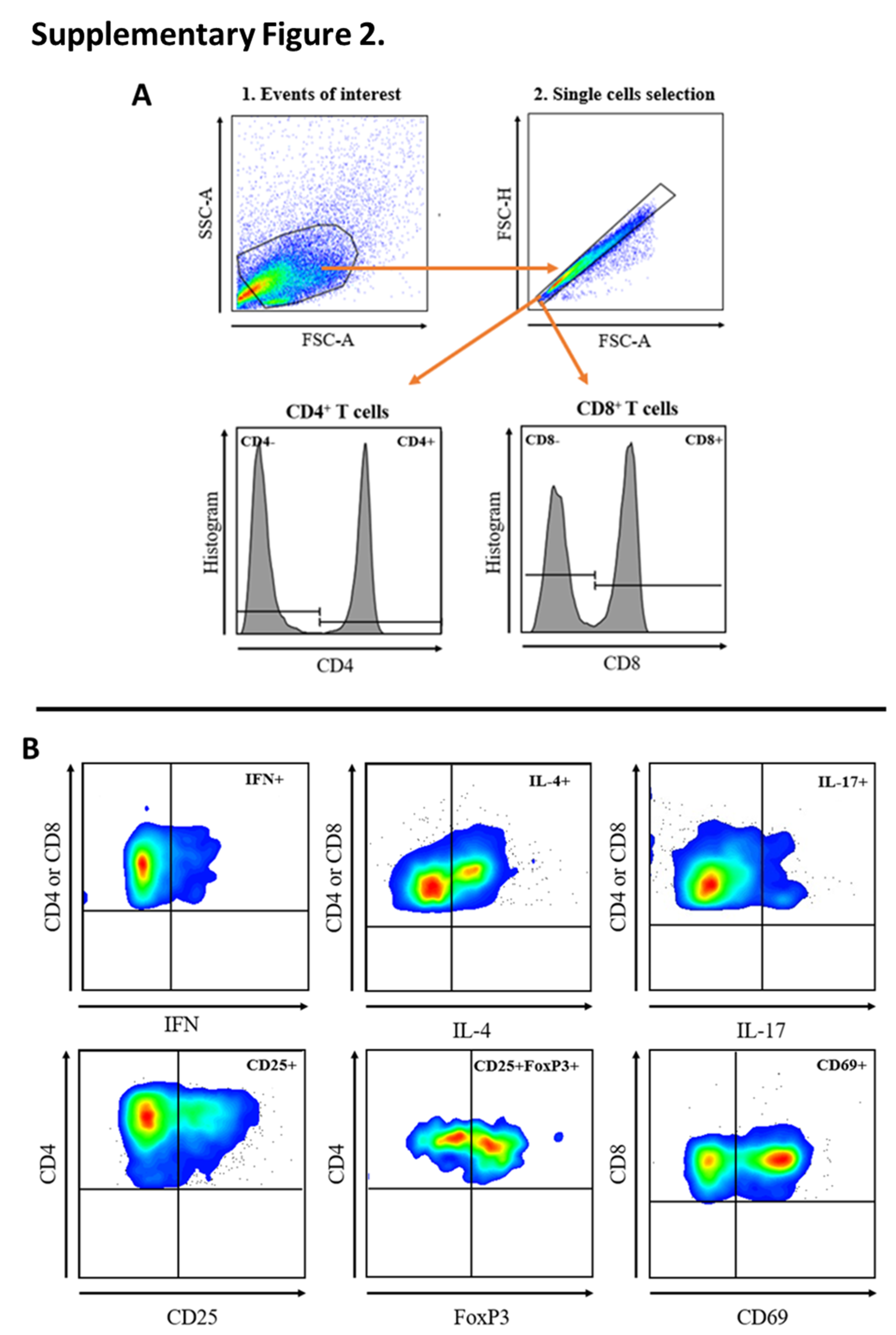

Supplement: Supplemental Material [file KVIR_A_2329573_SM2101.zip › Supplementary Figure 2.tiff]

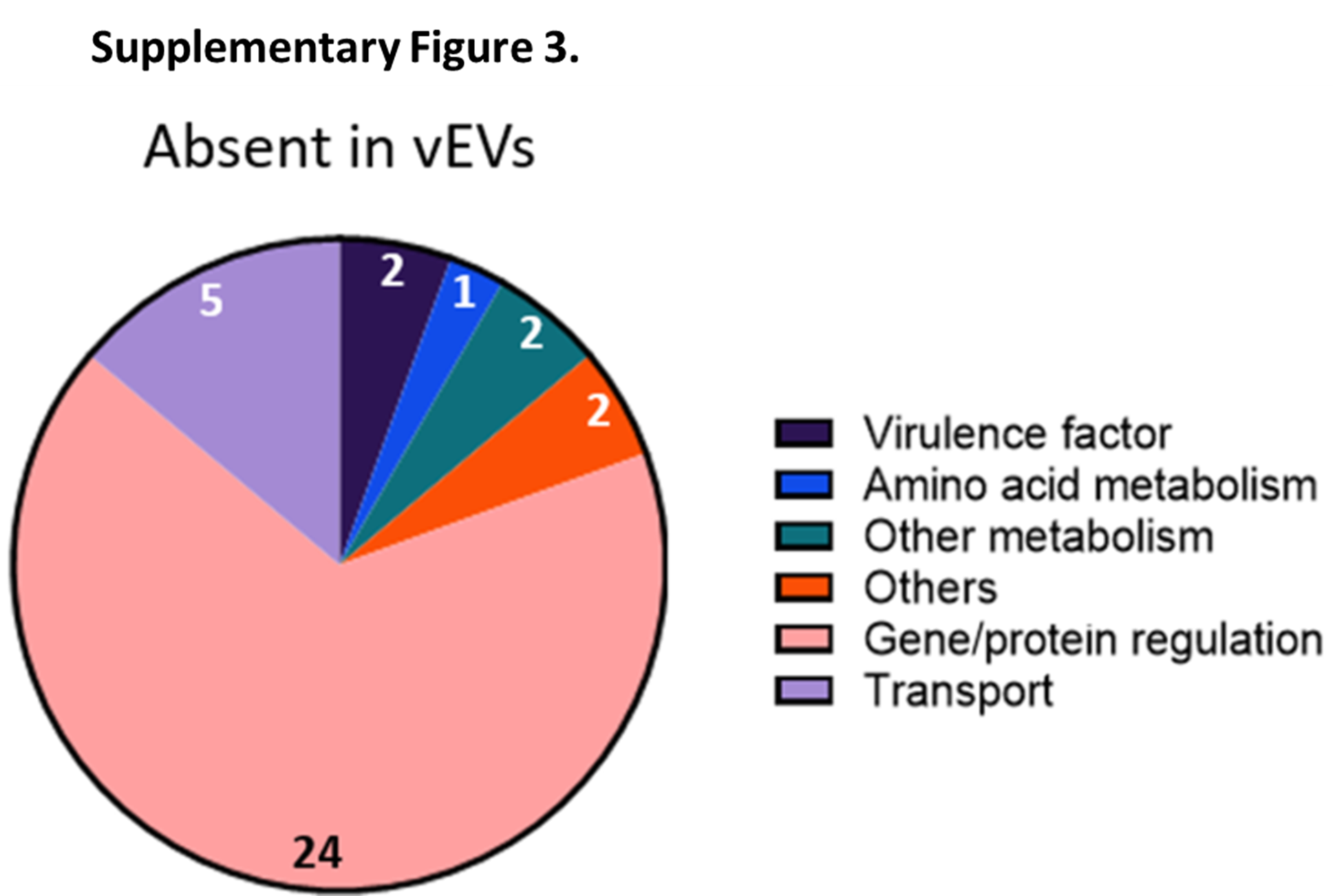

Supplement: Supplemental Material [file KVIR_A_2329573_SM2101.zip › Supplementary Figure 3.tiff]
